# Supplementary material for: Structural aging of human neurons is opposite of the changes in schizophrenia
Source: PLoS One. 2023 Jun 23;18(6):e0287646. doi: 10.1371/journal.pone.0287646 (PMC10289376; doi:10.1371/journal.pone.0287646)
Supplement: S1 Fig — A–N. Rendering of three-dimensional images of tissue structures and their Cartesian coordinate models. Renderings and models are viewed from nearly the same direction. The pial surface is toward the top. Three-dimensional images were rendered with the scatter HQ algorithm of the VG Studio software. Models were drawn with the MCTrace software. Model constituents are color-coded. Nodes composing each constituent are indicated with octagons. Dots indicate somata nodes. Scale bars: 10 μm. (PDF) [file pone.0287646.s001.pdf]

A **B**

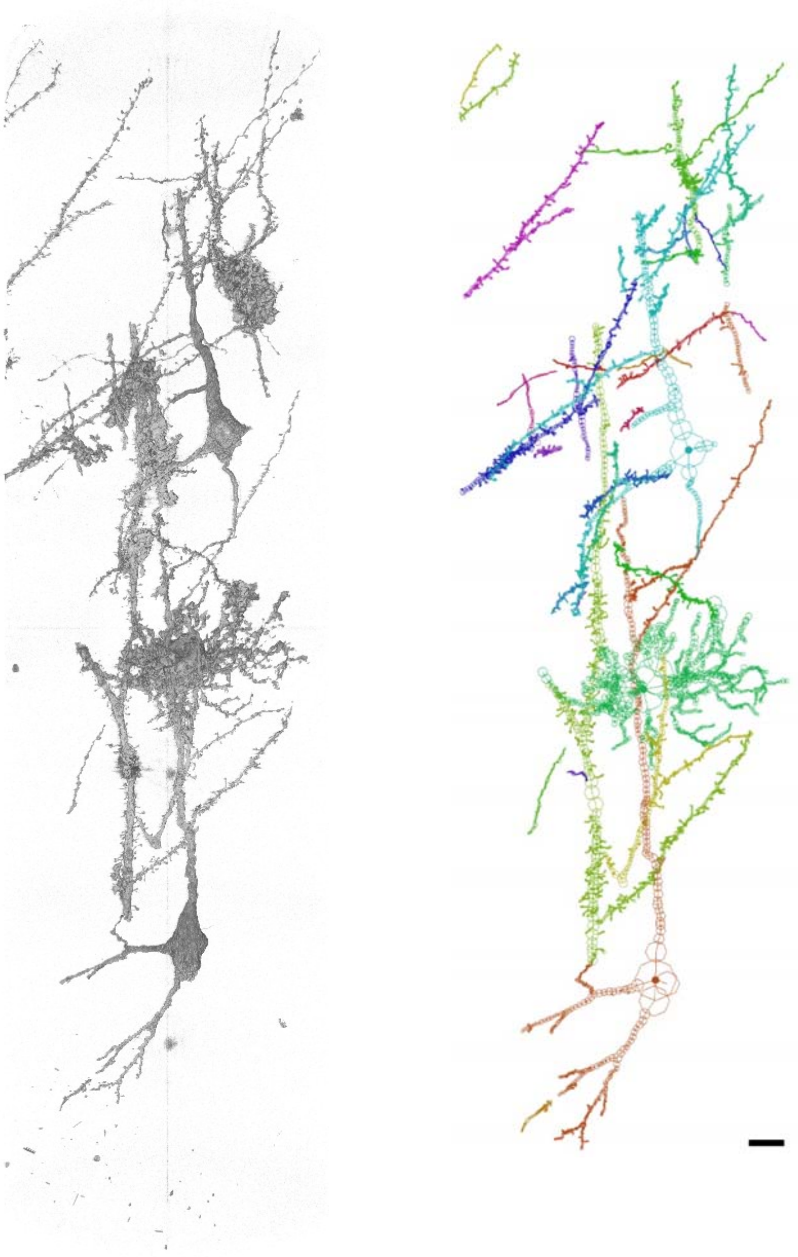

S1 Fig. Rendering of three-dimensional images of tissue structures and their Cartesian coordinate models. Renderings and models are viewed from nearly the same direction. The pial surface is toward the top. Three-dimensional images were rendered with the scatter HQ algorithm of the VG Studio software. Models were drawn with the MCTrace software. Model constituents are color-coded. Nodes composing each constituent are indicated with octagons. Dots indicate somata nodes. Scale bars: 10 μm. (**A**) Rendering of the S5A dataset of the schizophrenia S5 case. Voxel values of 60–800 are rendered. (**B**) Cartesian coordinate model of S5A.

C D

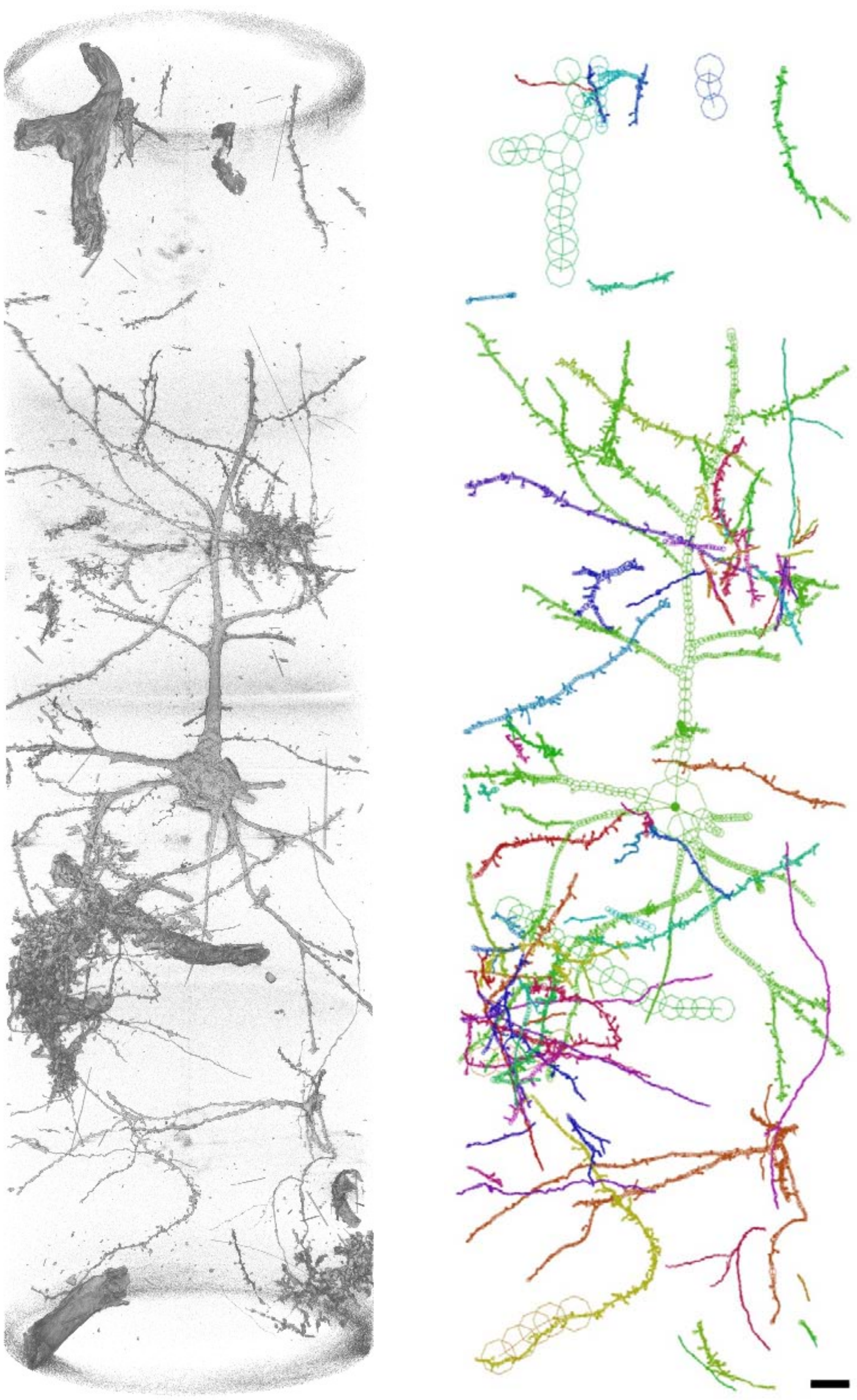

S1 Fig (cont'd). Rendering of three-dimensional images of tissue structures and their Cartesian coordinate models. Scale bars: 10 μm. (**C**) Rendering of the S6A dataset of the schizophrenia S6 case. Voxel values of 60–800 are rendered. (**D**) Model of S6A.

E **F**

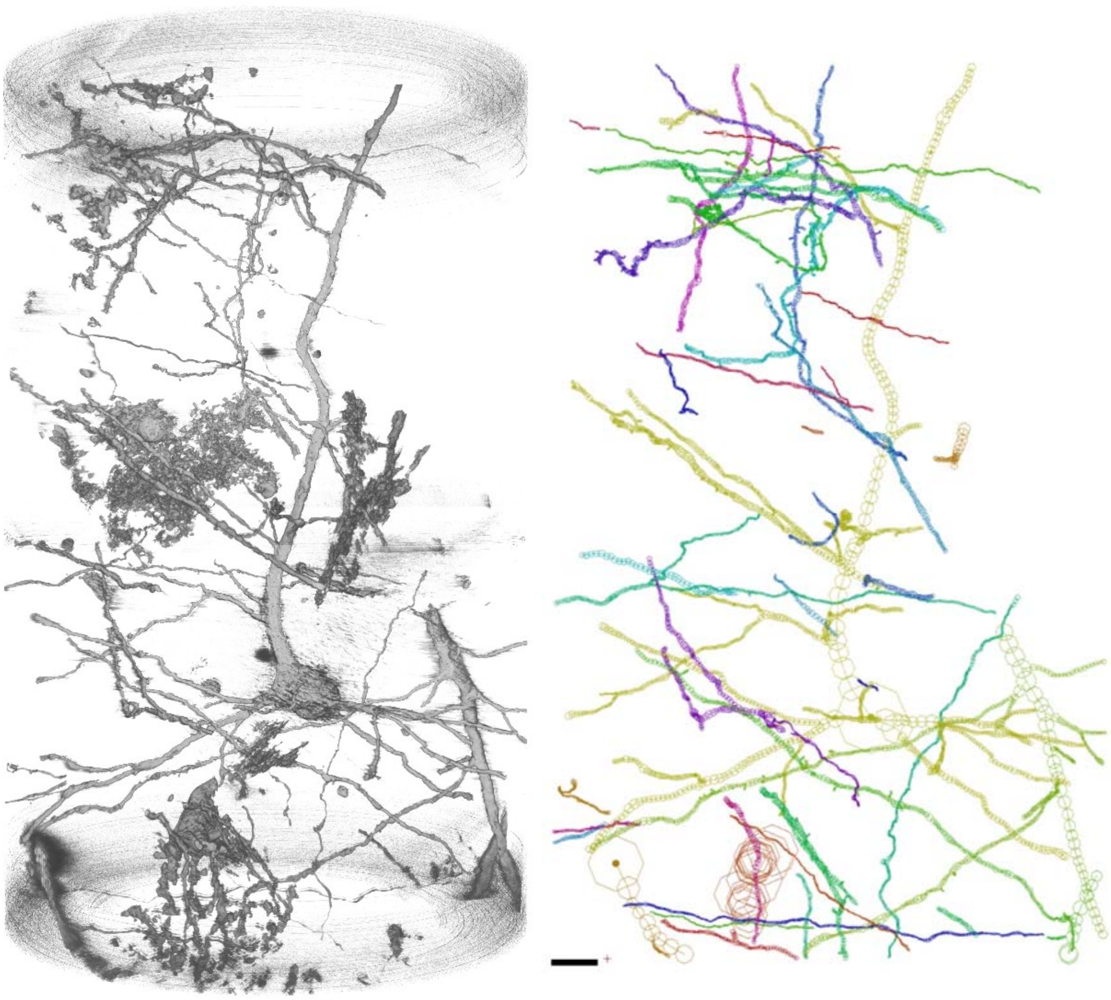

S1 Fig (cont'd). Rendering of three-dimensional images of tissue structures and their Cartesian coordinate models. Scale bars: 10 μm. (**E**) Rendering of the S7A dataset of the schizophrenia S7 case. Voxel values of 60–700 are rendered. (**F**) Model of S7A.

G **H**

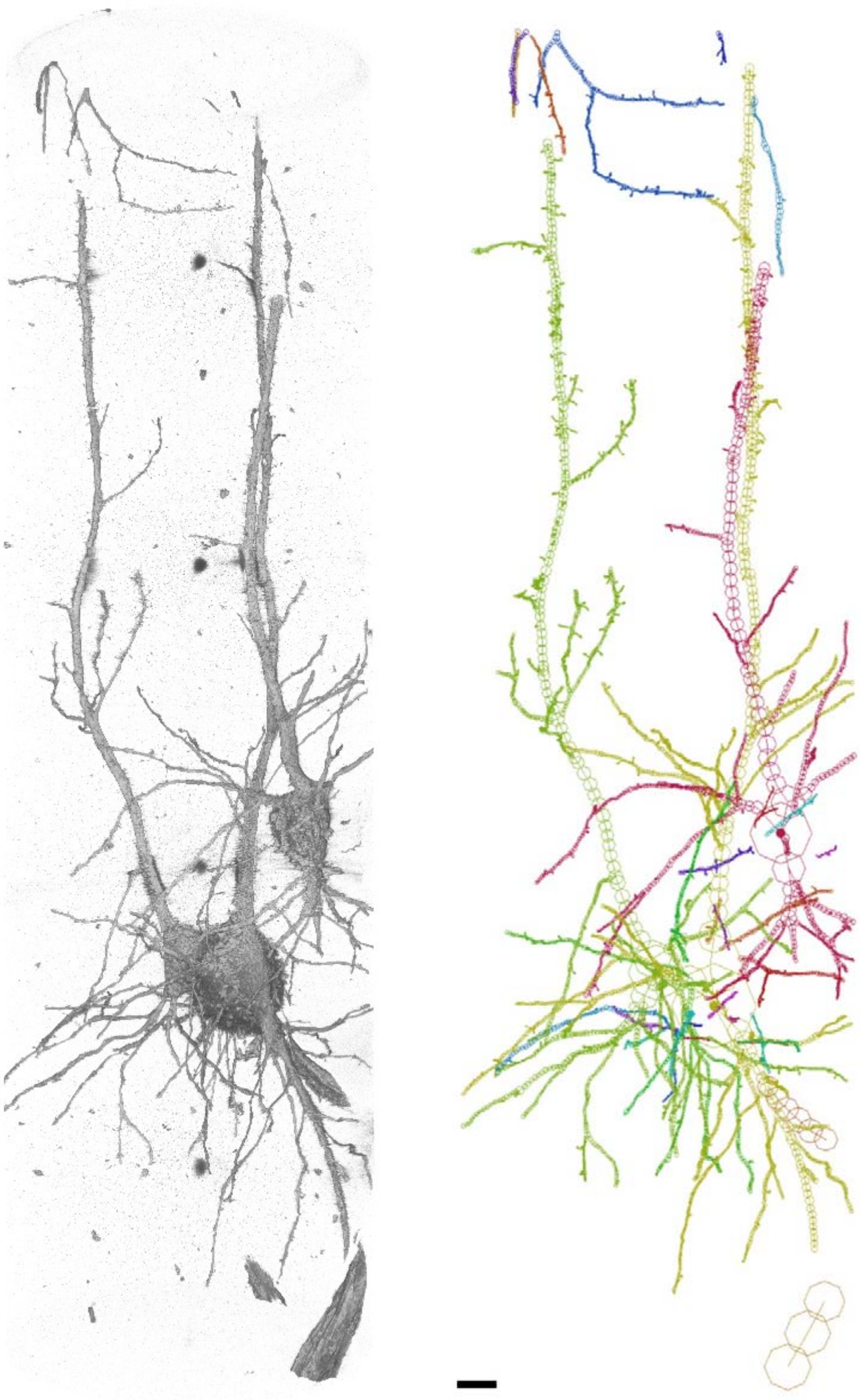

S1 Fig (cont'd). Rendering of three-dimensional images of tissue structures and their Cartesian coordinate models. Scale bars: 10 μm. (**G**) Rendering of the N5A dataset of the schizophrenia N5 case. Voxel values of 60–800 are rendered. (**H**) Model of N5A.

I **J**

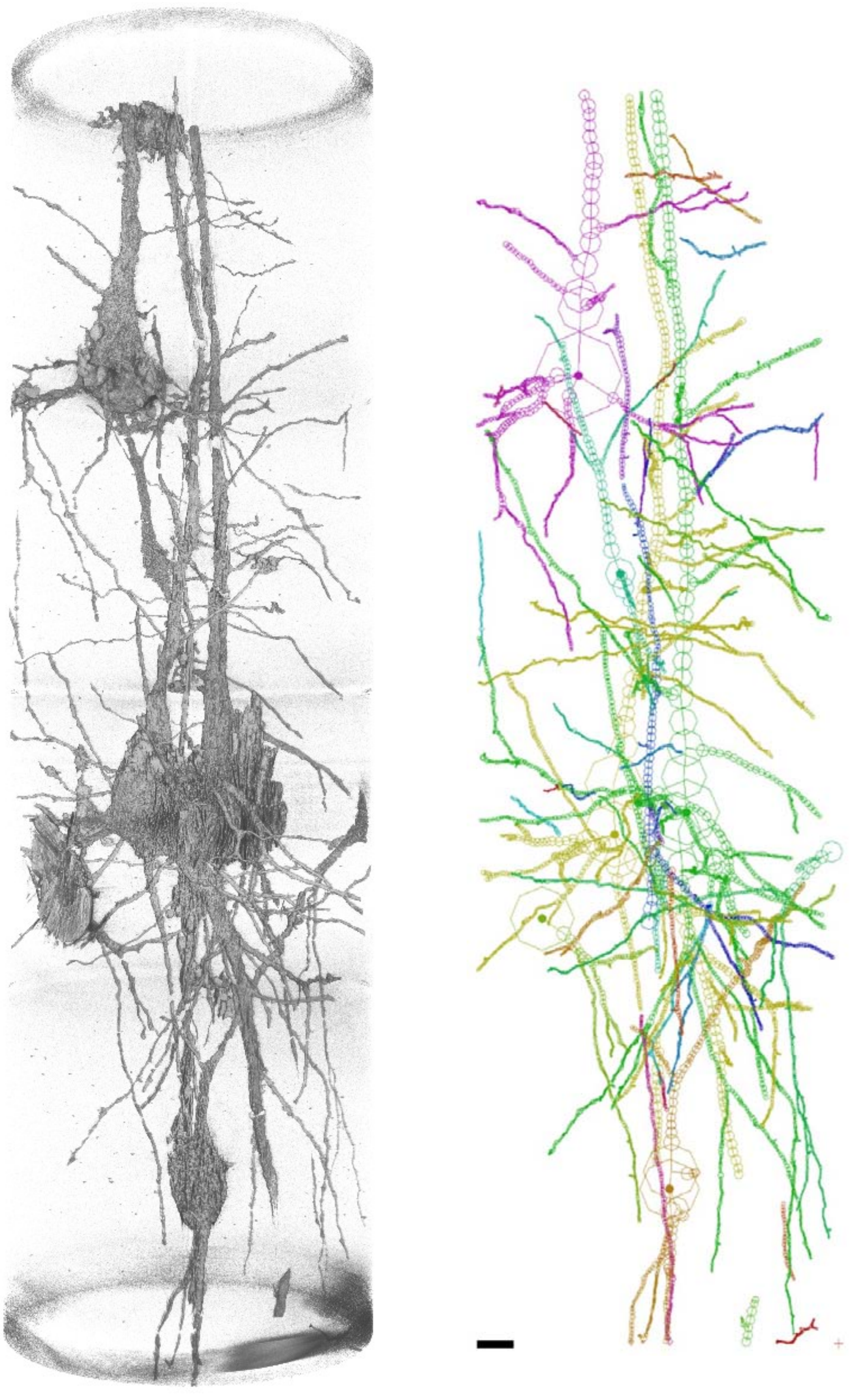

S1 Fig (cont'd). Rendering of three-dimensional images of tissue structures and their Cartesian coordinate models. Scale bars: 10 μm. (**I**) Rendering of the N6A dataset of the schizophrenia N6 case. Voxel values of 60–800 are rendered. (**J**) Model of N6A.

K **L**

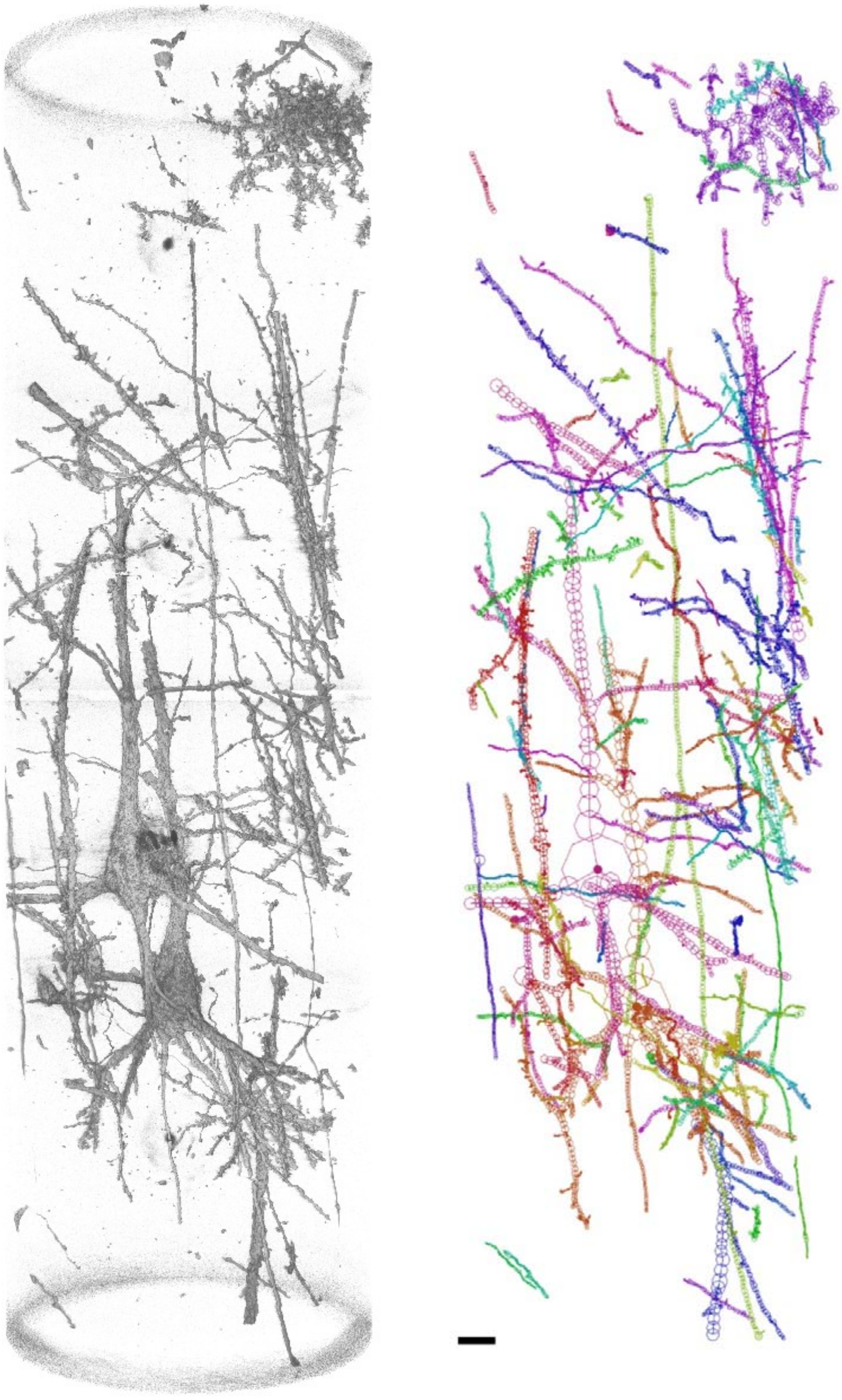

S1 Fig (cont'd). Rendering of three-dimensional images of tissue structures and their Cartesian coordinate models. Scale bars: 10 μm. (**K**) Rendering of the N7A dataset of the schizophrenia N7 case. Voxel values of 60–800 are rendered. (**L**) Model of N7A.

M **N**

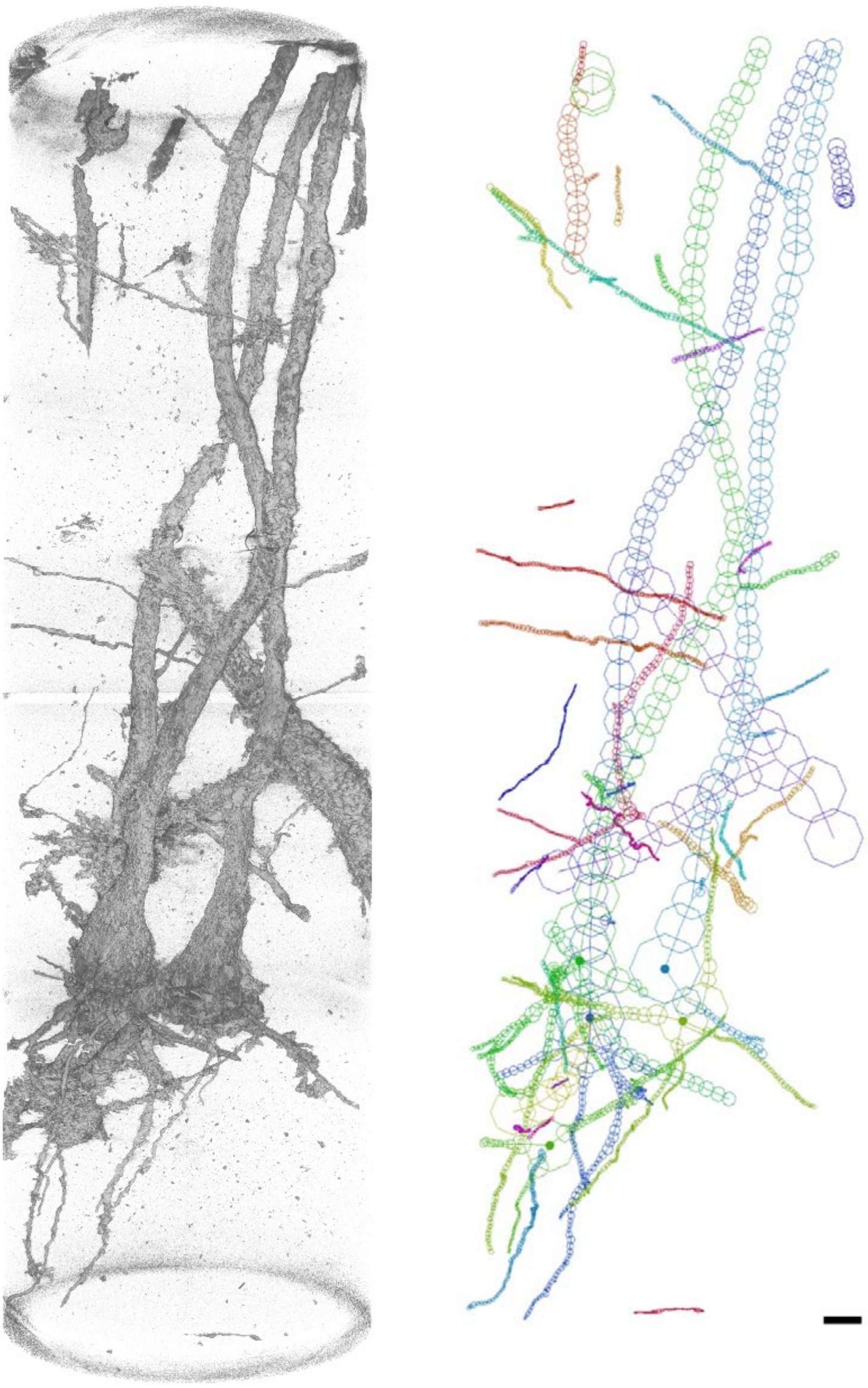

S1 Fig (cont'd). Rendering of three-dimensional images of tissue structures and their Cartesian coordinate models. Scale bars: 10 μm. (**M**) Rendering of the N8A dataset of the schizophrenia N8 case. Voxel values of 60–800 are rendered. (**N**) Model of N8A.
