# Supplementary material for: Structural aging of human neurons is opposite of the changes in schizophrenia
Source: PLoS One. 2023 Jun 23;18(6):e0287646. doi: 10.1371/journal.pone.0287646 (PMC10289376; doi:10.1371/journal.pone.0287646)
Supplement: S2 Fig — A–T. Cartesian coordinate models of schizophrenia case structures. The pial surface is toward the top. Models were drawn with the MCTrace software. Constituents of the models are color-coded. Nodes composing each constituent are indicated with octagons. Dots indicate somata nodes. Scale bars: 10 μm. (PDF) [file pone.0287646.s002.pdf]

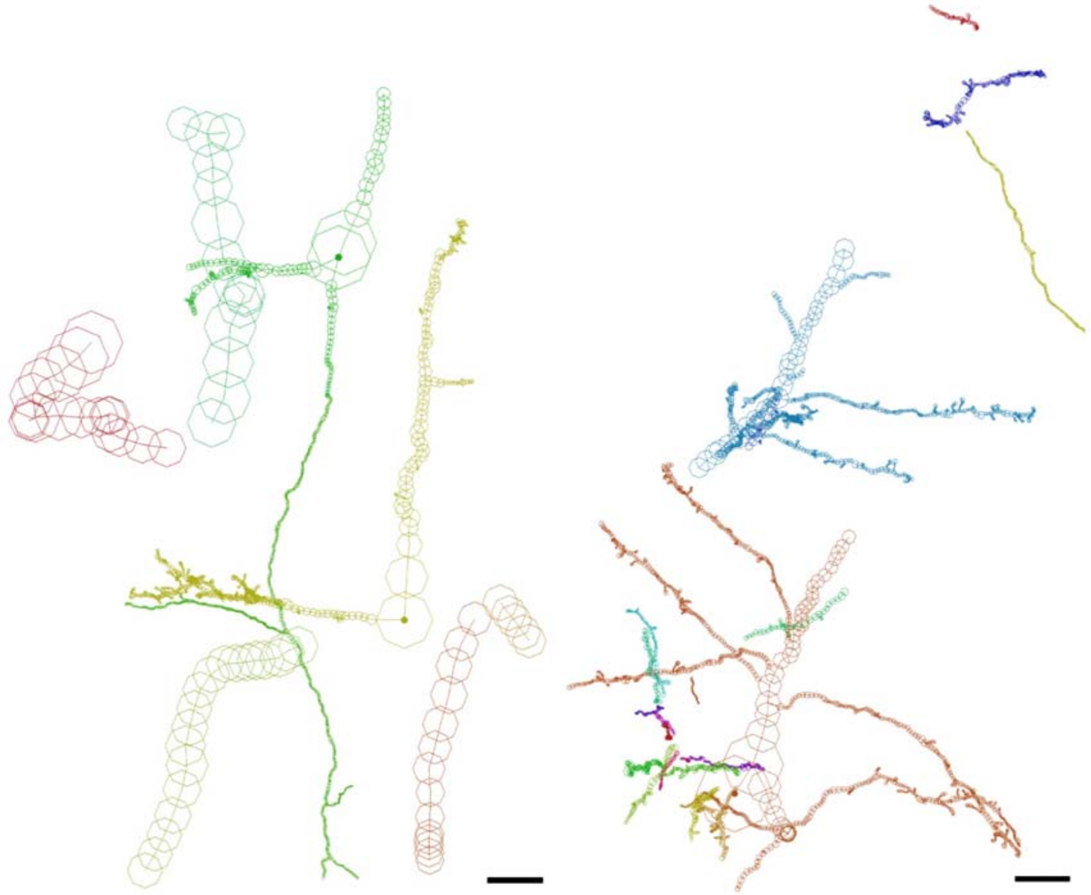

(**A**) S5B structure. (**B**) S5C structure.

S2 Fig. Cartesian coordinate models of schizophrenia case structures. The pial surface is toward the top. Models were drawn with the MCTrace software. Constituents of the models are color-coded. Nodes composing each constituent are indicated with octagons. Dots indicate somata nodes. Scale bars: 10 µm.

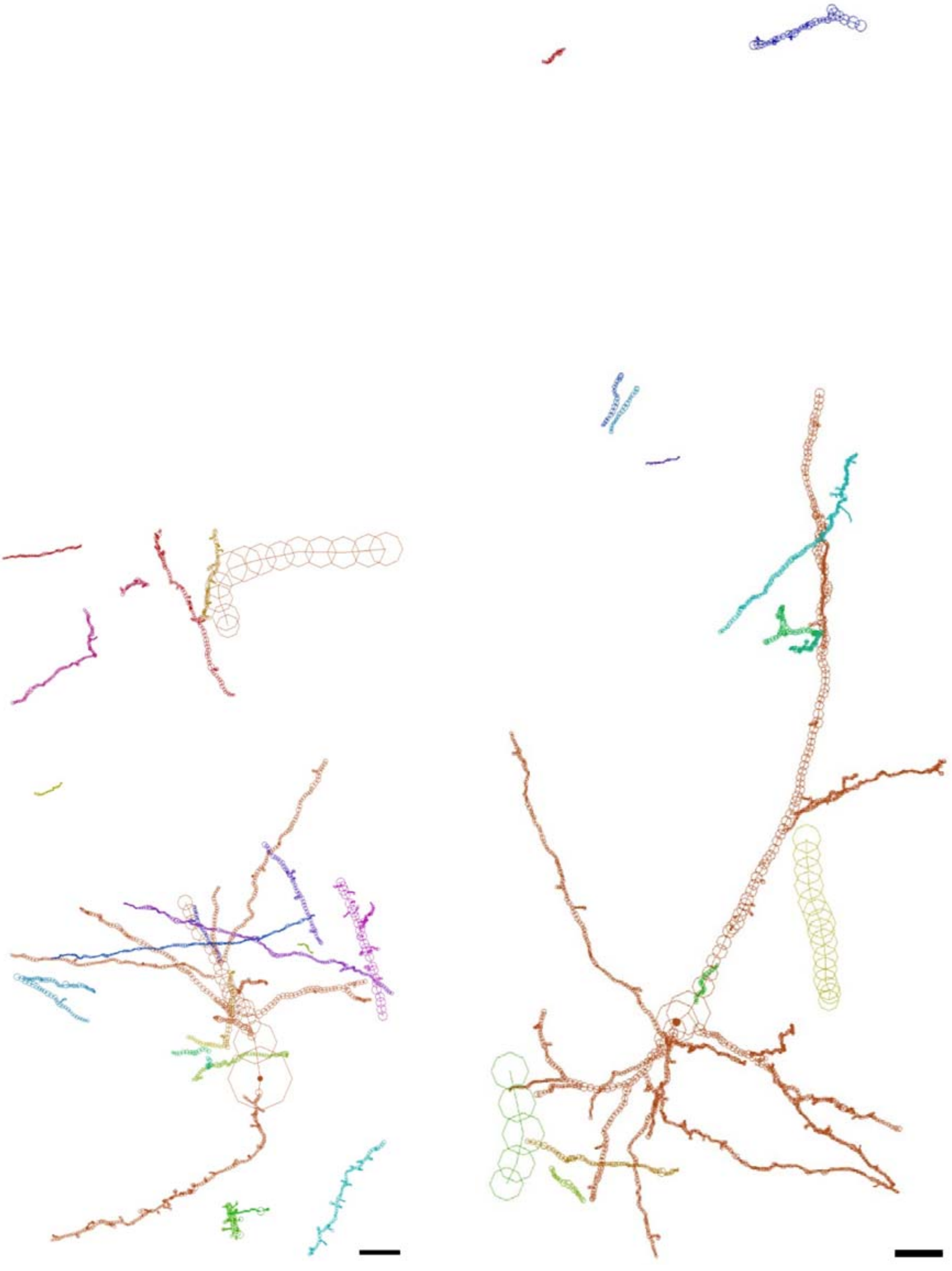

(**C**) S5D structure. (**D**) S5E structure.

S2 Fig (cont'd). Cartesian coordinate models of schizophrenia case structures. The pial surface is toward the top. The models were drawn with the MCTrace software. Constituents of the models are color-coded. Nodes composing each constituent are indicated with octagons. Dots indicate somata nodes. Scale bars: 10 μm.

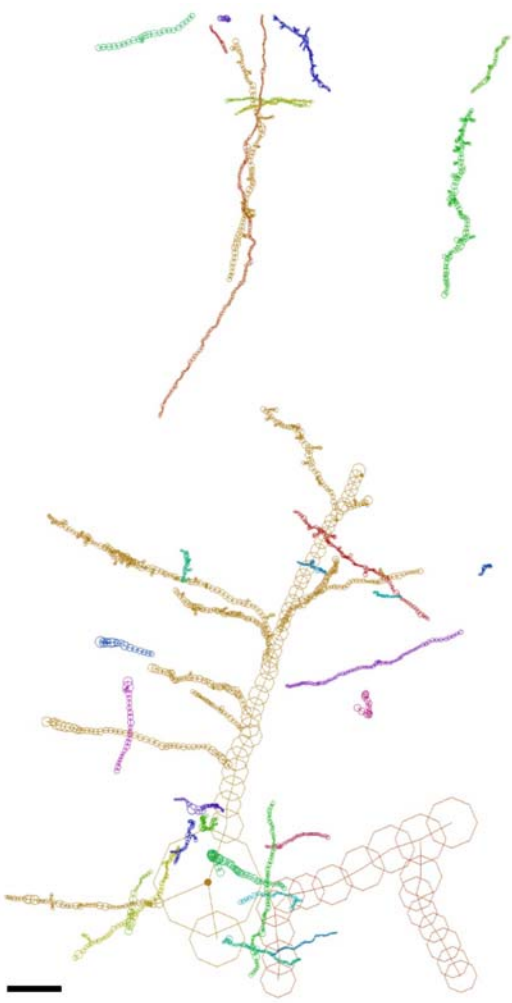

(**E**) S5F structure.

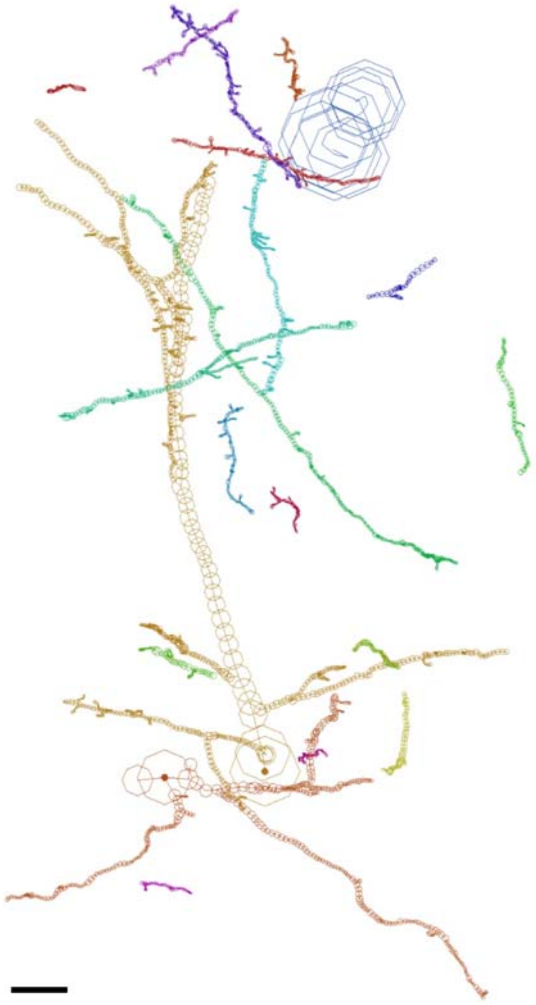

(**F**) S5G structure.

S2 Fig (cont'd). Cartesian coordinate models of schizophrenia case structures. The pial surface is toward the top. The models were drawn with the MCTrace software. Constituents of the models are color-coded. Nodes composing each constituent are indicated with octagons. Dots indicate somata nodes. Scale bars: 10 μm.

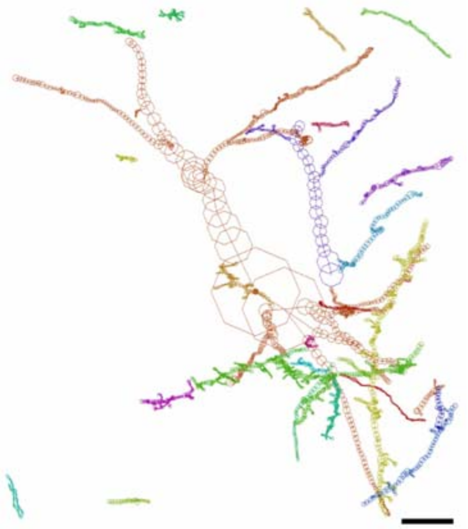

(**G**) S5H structure.

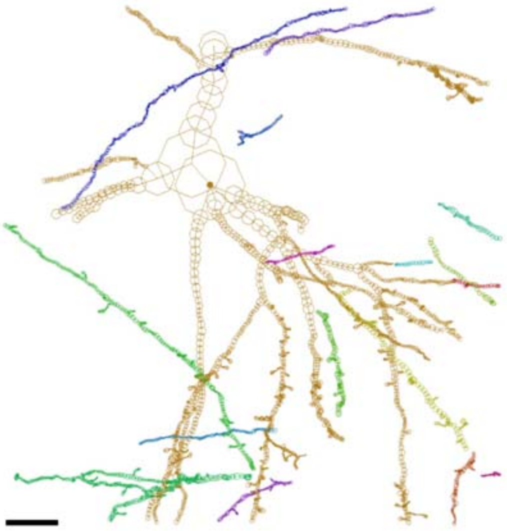

(**H**) S5I structure.

S2 Fig (cont'd). Cartesian coordinate models of schizophrenia case structures. The pial surface is toward the top. The models were drawn with the MCTrace software. Constituents of the models are color-coded. Nodes composing each constituent are indicated with octagons. Dots indicate somata nodes. Scale bars: 10 µm.

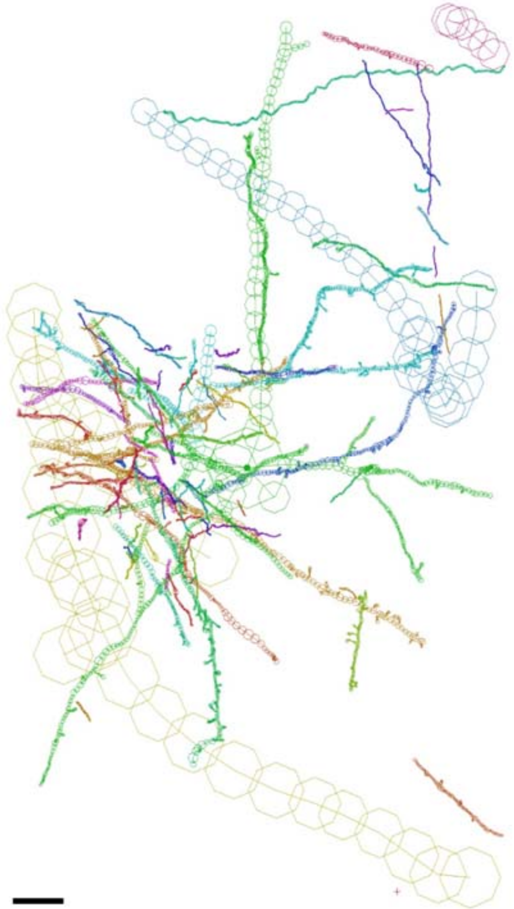

(**I**) S6B structure.

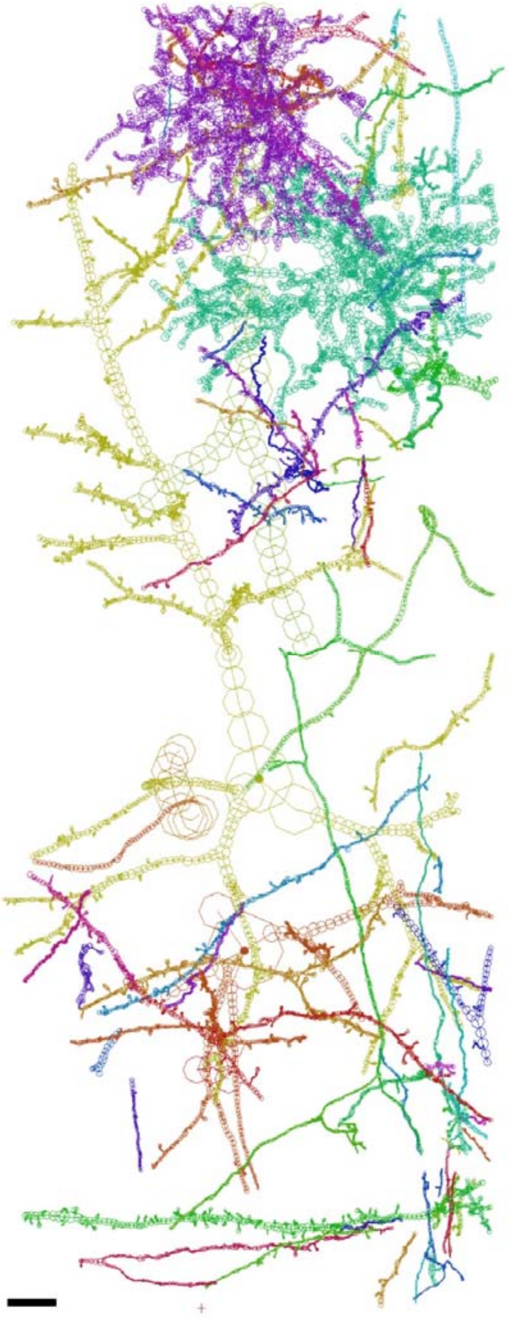

(**J**) S6C structure.

S2 Fig (cont'd). Cartesian coordinate models of schizophrenia case structures. The pial surface is toward the top. The models were drawn with the MCTrace software. Constituents of the models are color-coded. Nodes composing each constituent are indicated with octagons. Dots indicate somata nodes. Scale bars: 10 μm.

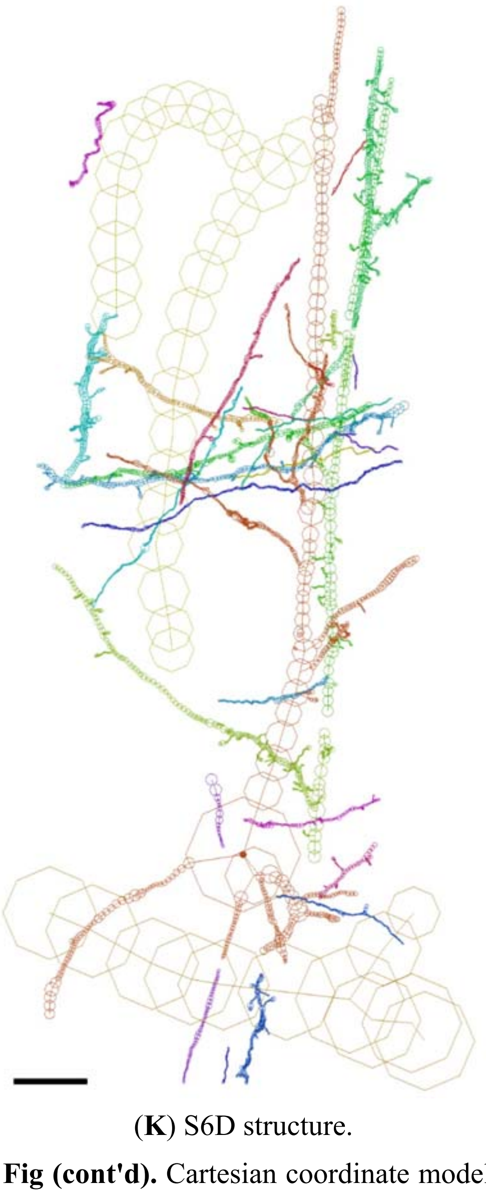

(**K**) S6D structure.

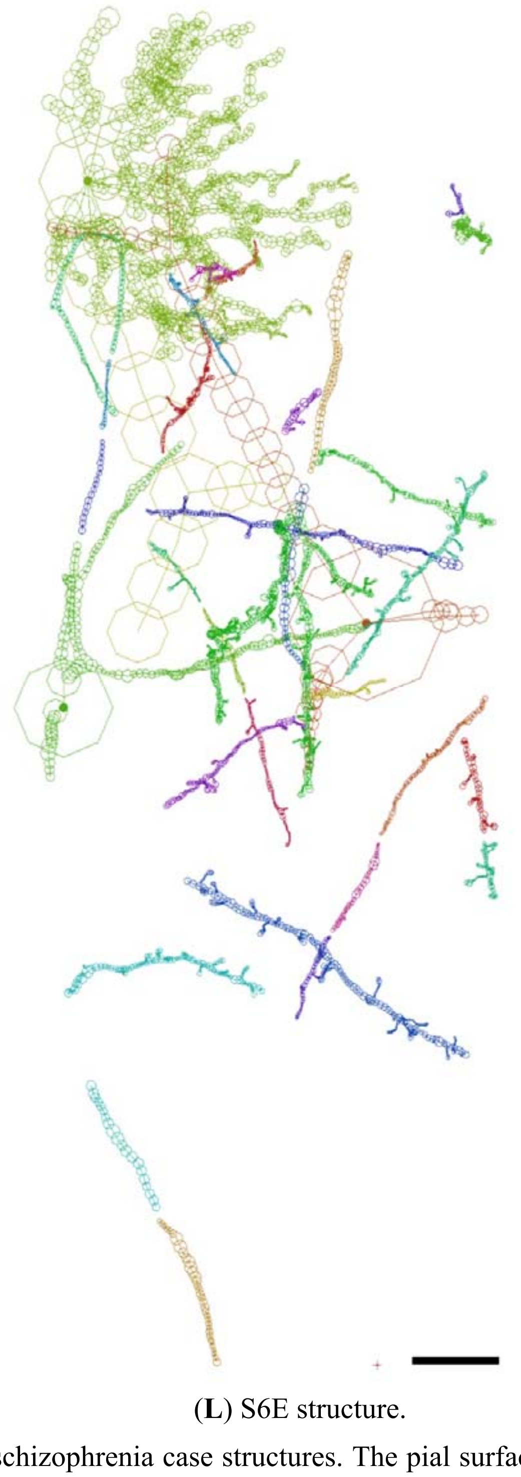

(**L**) S6E structure.

S2 Fig (cont'd). Cartesian coordinate models of schizophrenia case structures. The pial surface is toward the top. The models were drawn with the MCTrace software. Constituents of the models are color-coded. Nodes composing each constituent are indicated with octagons. Dots indicate somata nodes. Scale bars: 10 µm.

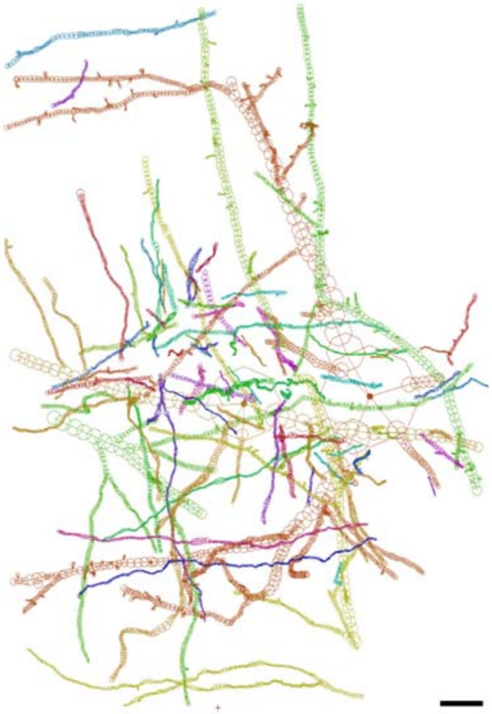

(**M**) S7B structure.

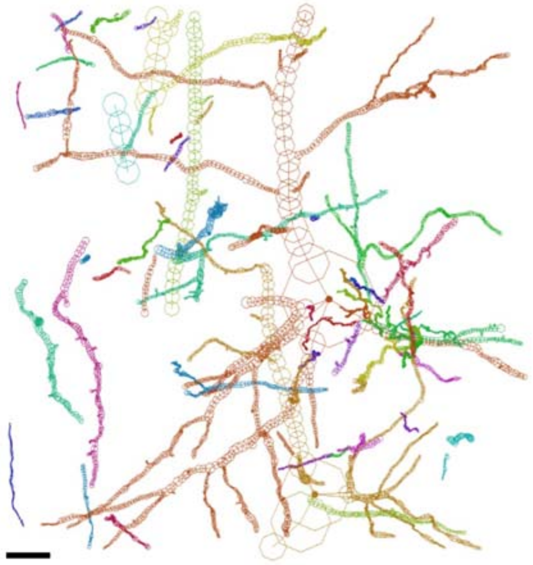

(**N**) S7C structure.

S2 Fig (cont'd). Cartesian coordinate models of schizophrenia case structures. The pial surface is toward the top. The models were drawn with the MCTrace software. Constituents of the models are color-coded. Nodes composing each constituent are indicated with octagons. Dots indicate somata nodes. Scale bars: 10 μm.

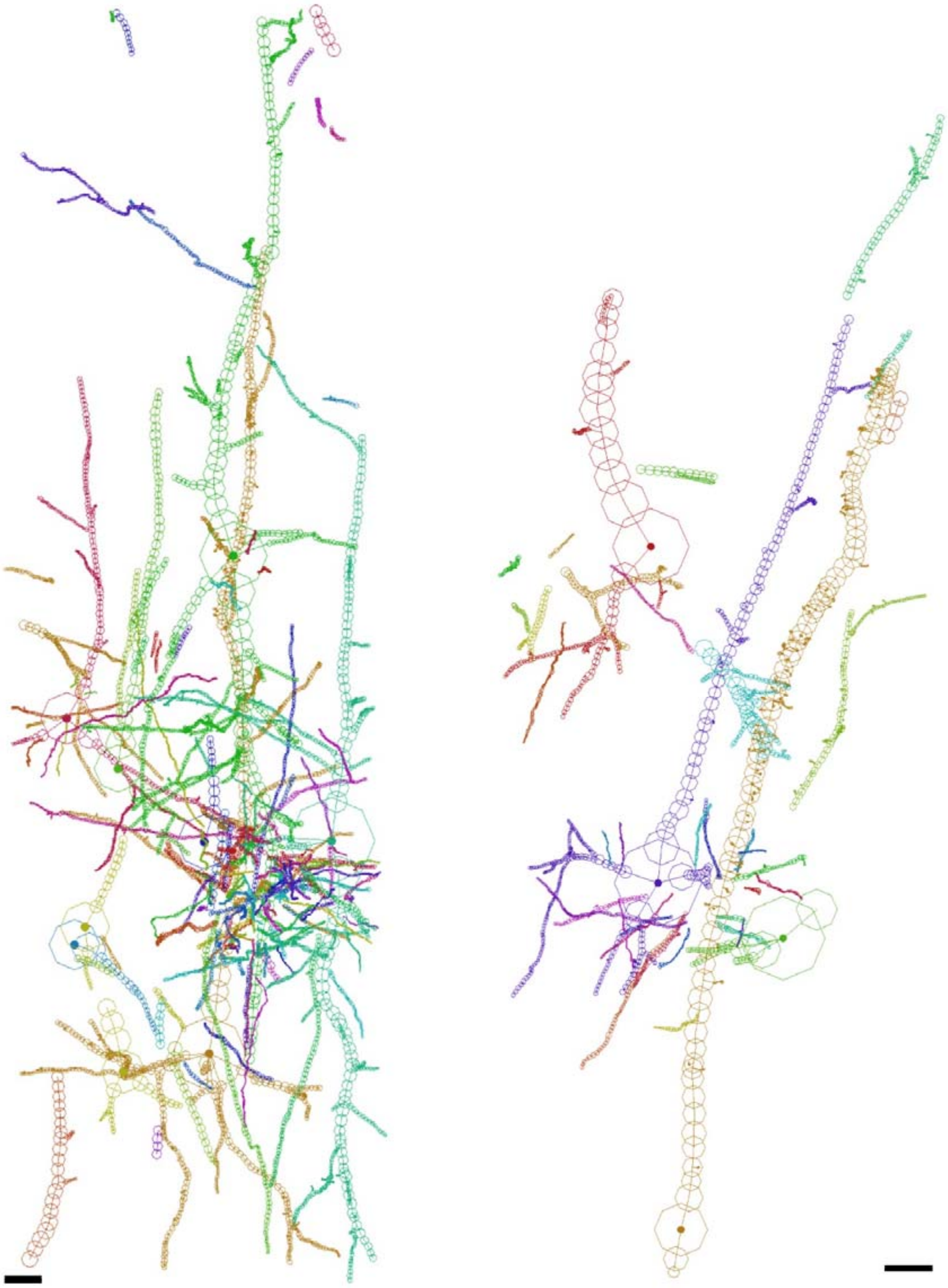

(**O**) S7D structure. (**P**) S7E structure.

S2 Fig (cont'd). Cartesian coordinate models of schizophrenia case structures. The pial surface is toward the top. The models were drawn with the MCTrace software. Constituents of the models are color-coded. Nodes composing each constituent are indicated with octagons. Dots indicate somata nodes. Scale bars: 10 μm.

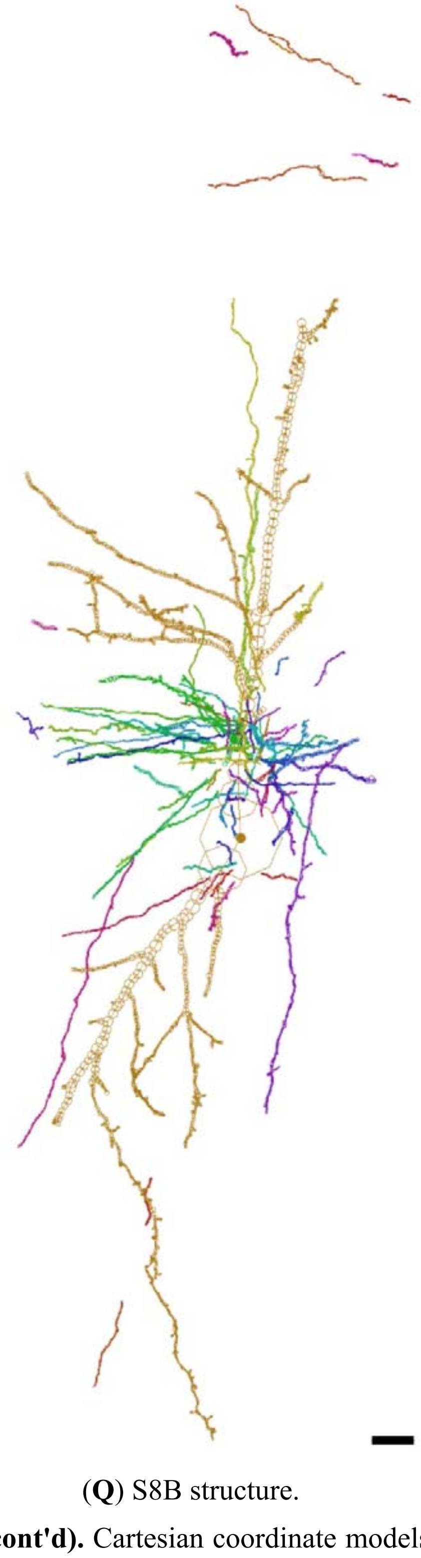

(**Q**) S8B structure.

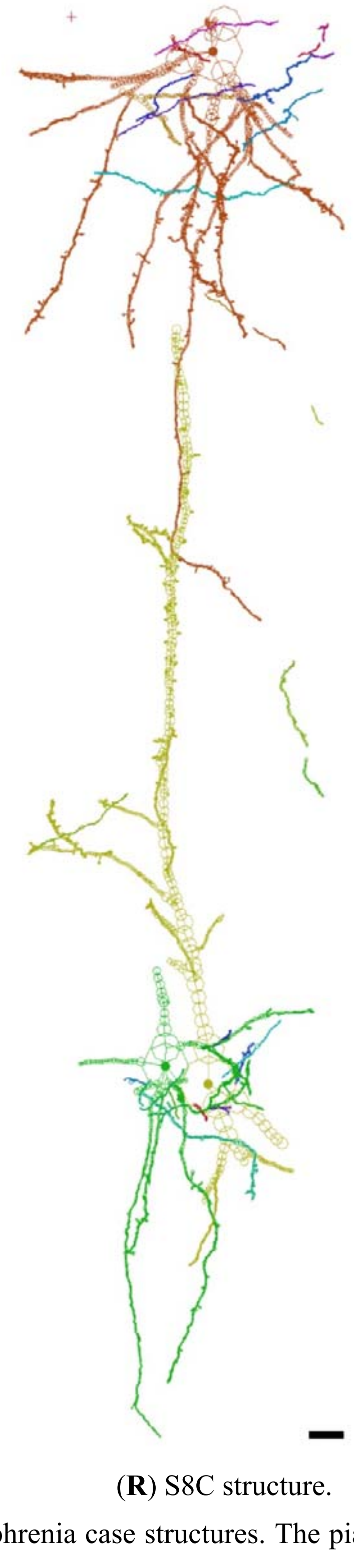

(**R**) S8C structure.

S2 Fig (cont'd). Cartesian coordinate models of schizophrenia case structures. The pial surface is toward the top. The models were drawn with the MCTrace software. Constituents of the models are color-coded. Nodes composing each constituent are indicated with octagons. Dots indicate somata nodes. Scale bars: 10 µm.

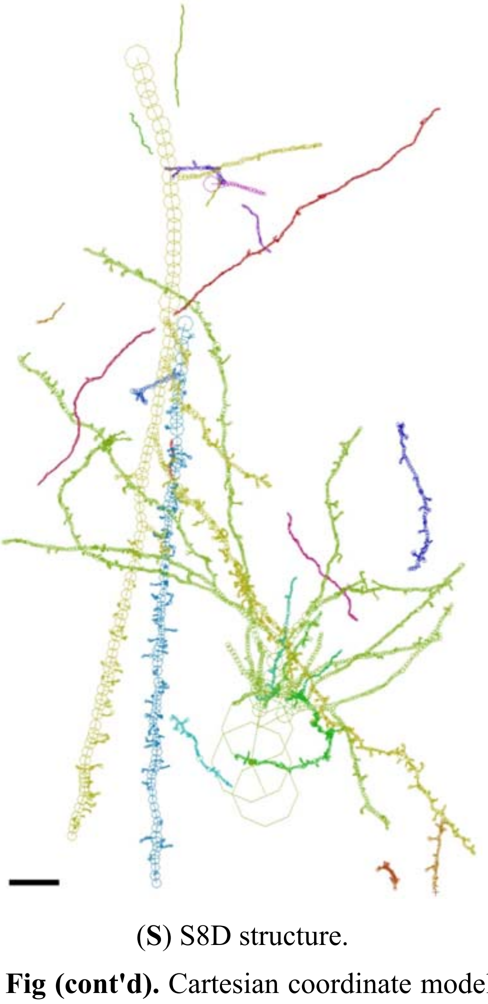

(**S**) S8D structure.

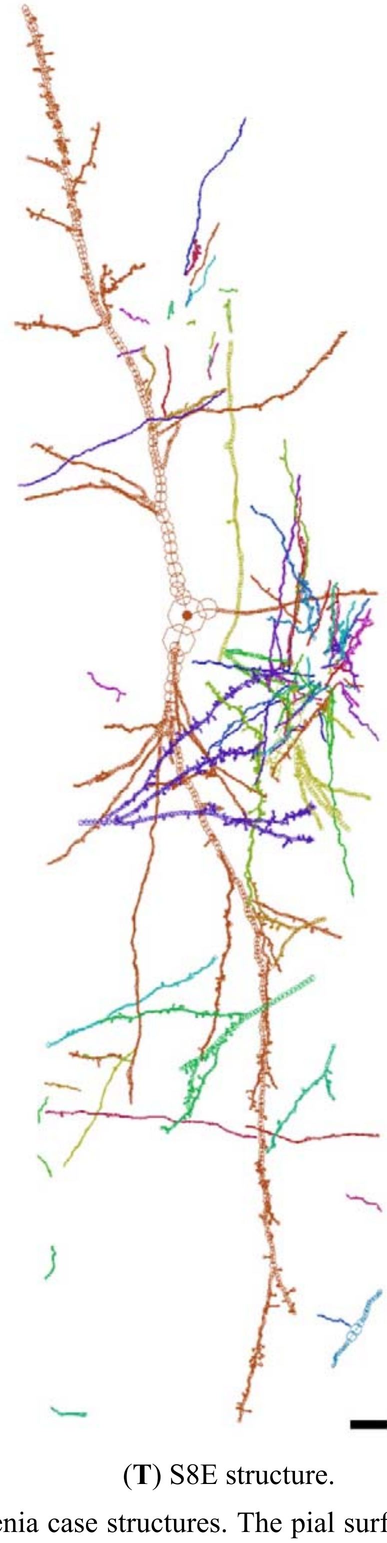

(**T**) S8E structure.

S2 Fig (cont'd). Cartesian coordinate models of schizophrenia case structures. The pial surface is toward the top. The models were drawn with the MCTrace software. Constituents of the models are color-coded. Nodes composing each constituent are indicated with octagons. Dots indicate somata nodes. Scale bars: 10 μm.
