# Supplementary material for: Structural aging of human neurons is opposite of the changes in schizophrenia
Source: PLoS One. 2023 Jun 23;18(6):e0287646. doi: 10.1371/journal.pone.0287646 (PMC10289376; doi:10.1371/journal.pone.0287646)
Supplement: S3 Fig — A–Z. Cartesian coordinate models of control case structures. The pial surface is toward the top. The models were drawn with the MCTrace software. Constituents of the models are color-coded. Nodes composing each constituent are indicated with octagons. Dots indicate somata nodes. Scale bars: 10 μm. (PDF) [file pone.0287646.s003.pdf]

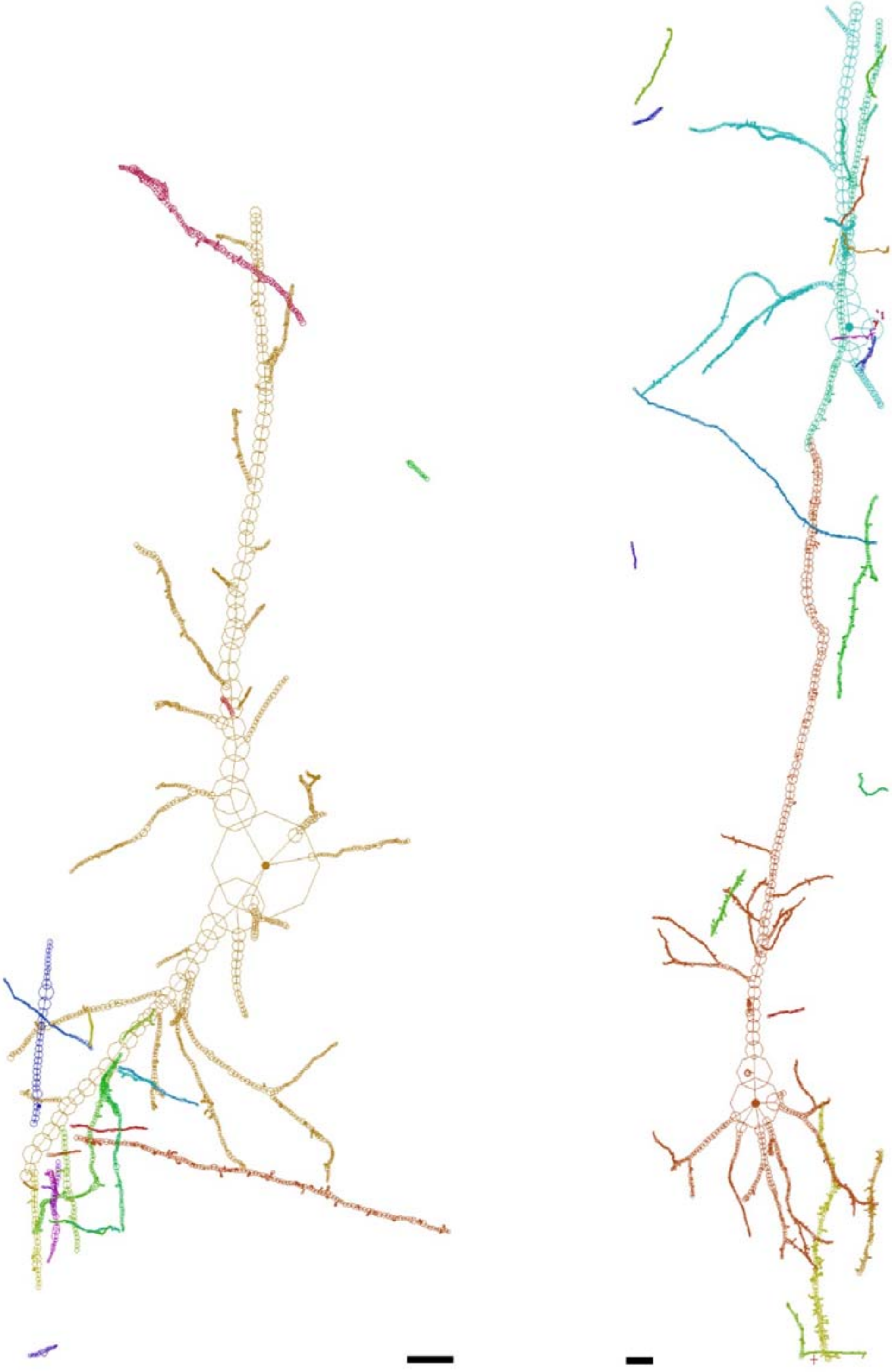

(**A**) N5B structure. (**B**) N5C structure.

S3 Fig. Cartesian coordinate models of control case structures. The pial surface is toward the top. The models were drawn with the MCTrace software. Constituents of the models are color-coded. Nodes composing each constituent are indicated with octagons. Dots indicate somata nodes. Scale bars: 10 µm.

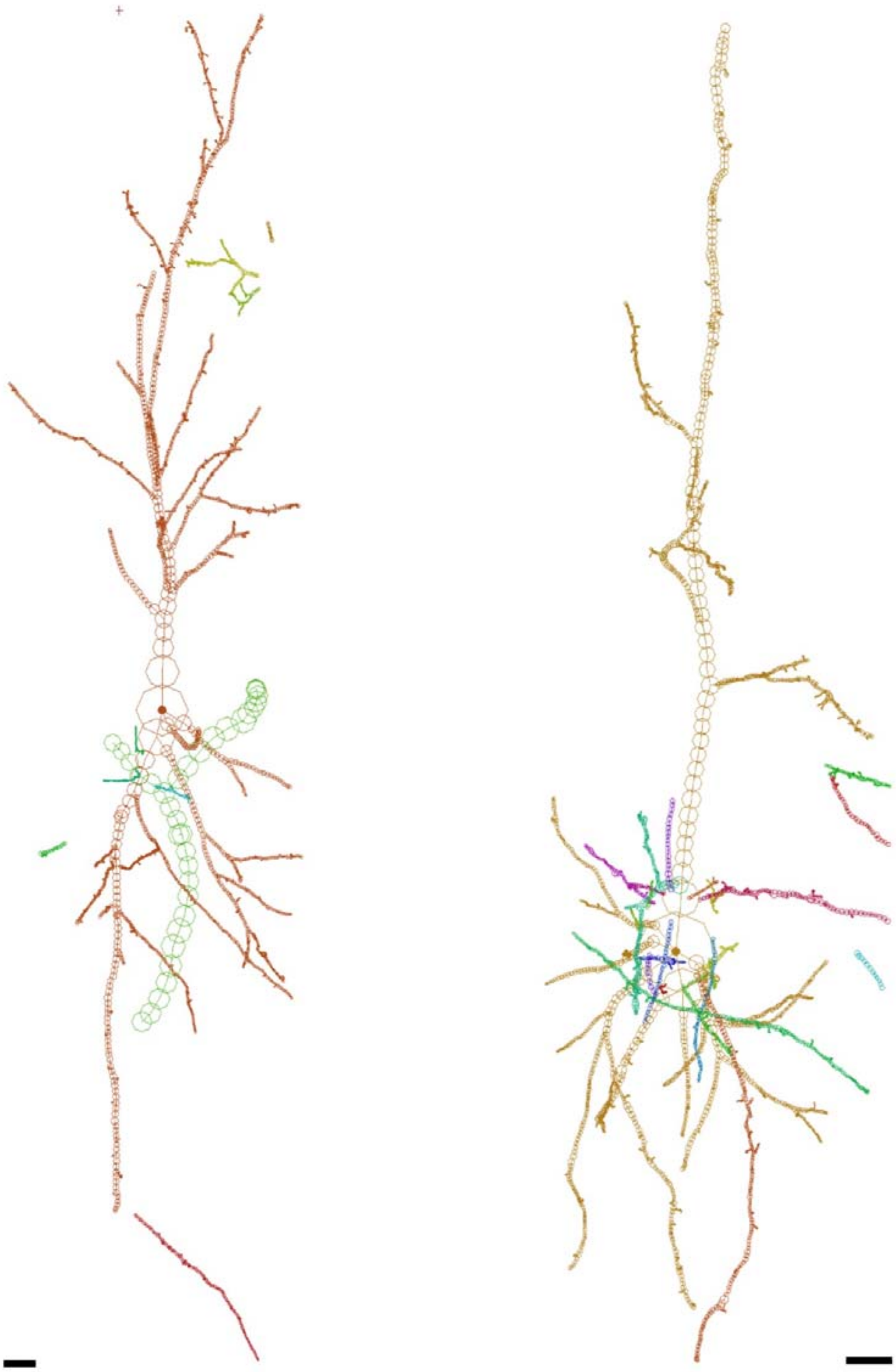

(**C**) N5D structure.

(**D**) N5E structure.

S3 Fig (cont'd). Cartesian coordinate models of control case structures. The pial surface is toward the top. The models were drawn with the MCTrace software. Constituents of the models are color-coded. Nodes composing each constituent are indicated with octagons. Dots indicate somata nodes. Scale bars: 10 µm.

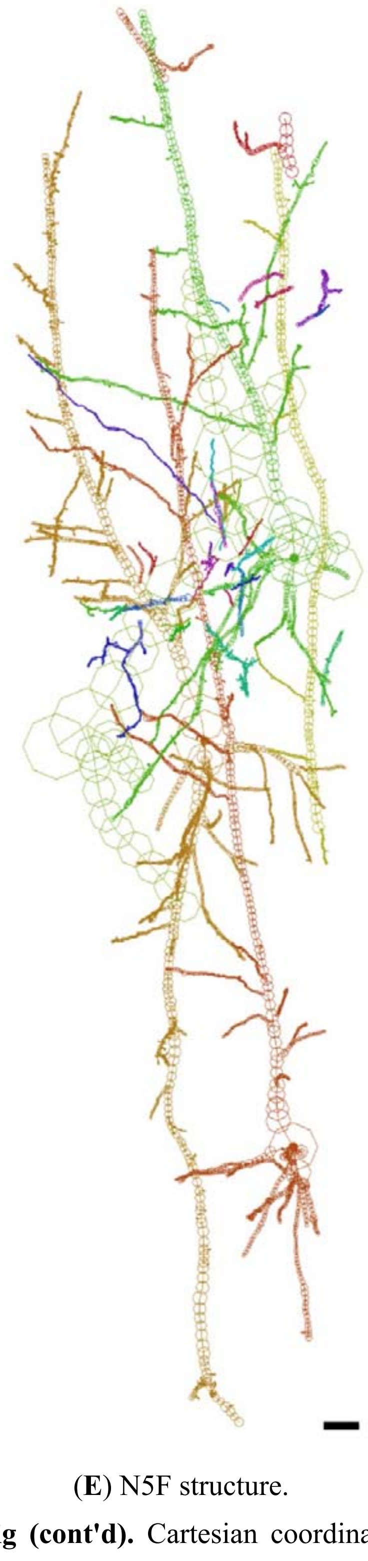

(**E**) N5F structure.

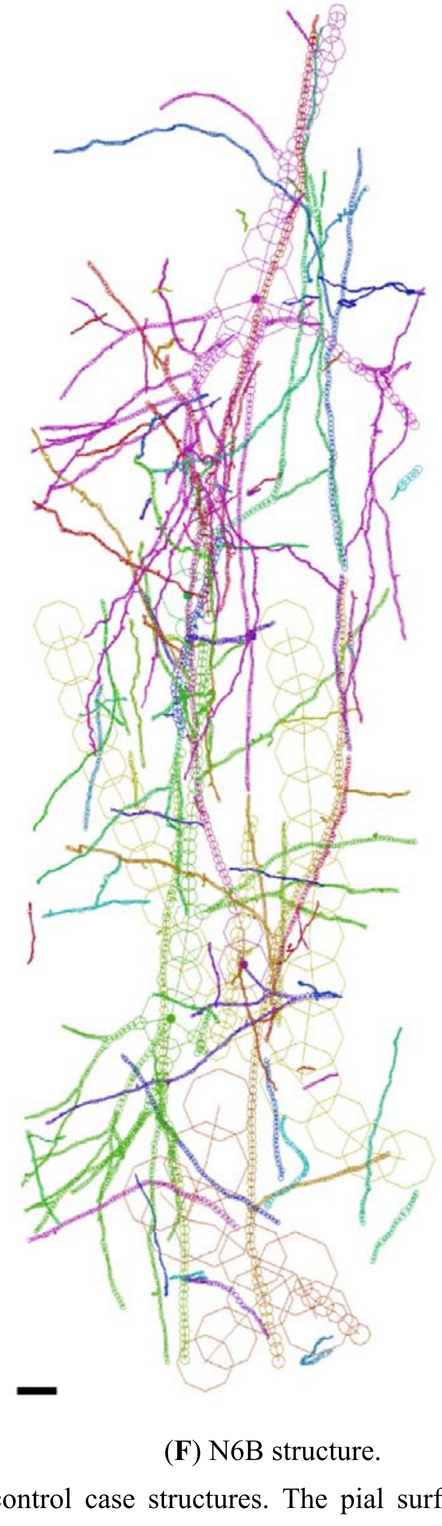

(**F**) N6B structure.

S3 Fig (cont'd). Cartesian coordinate models of control case structures. The pial surface is toward the top. The models were drawn with the MCTrace software. Constituents of the models are color-coded. Nodes composing each constituent are indicated with octagons. Dots indicate somata nodes. Scale bars: 10 μm.

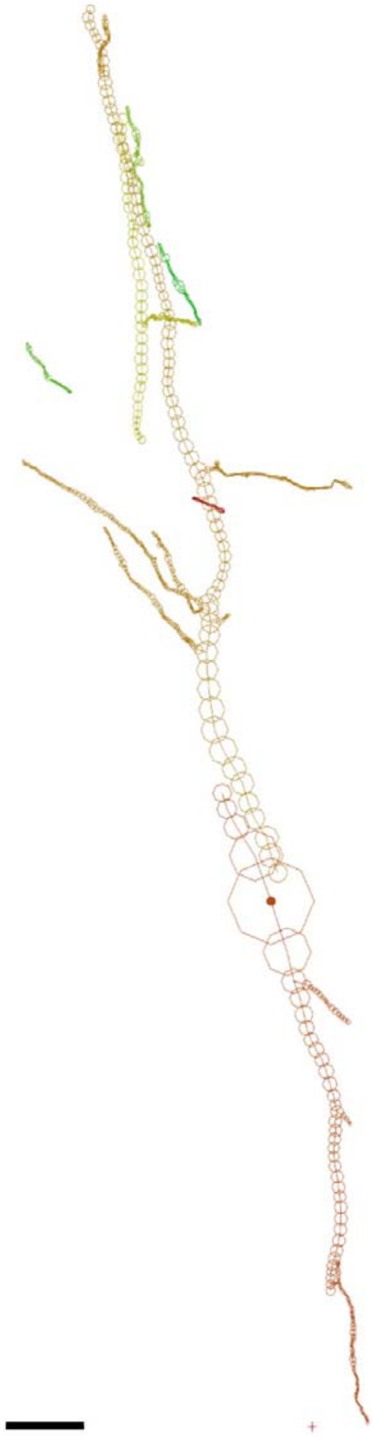

(**G**) N6C structure.

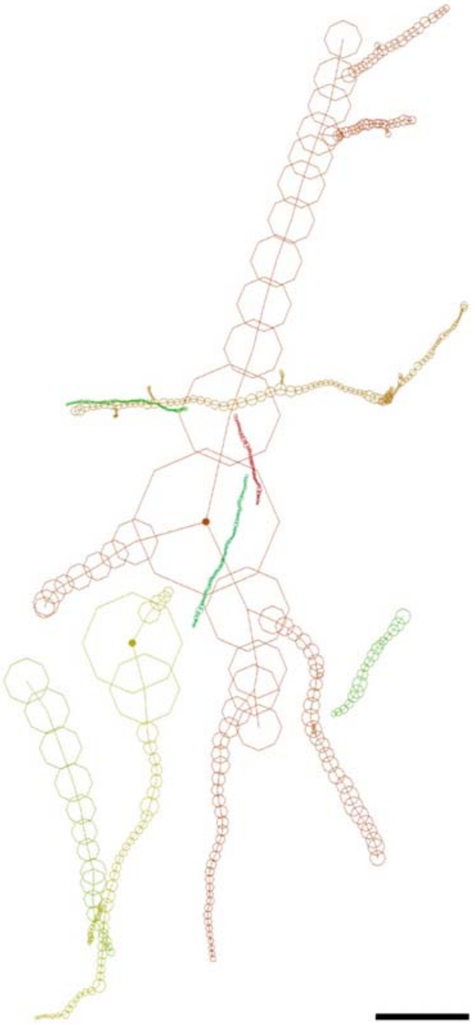

(**H**) N6D structure.

S3 Fig (cont'd). Cartesian coordinate models of control case structures. The pial surface is toward the top. The models were drawn with the MCTrace software. Constituents of the models are color-coded. Nodes composing each constituent are indicated with octagons. Dots indicate somata nodes. Scale bars: 10 μm.

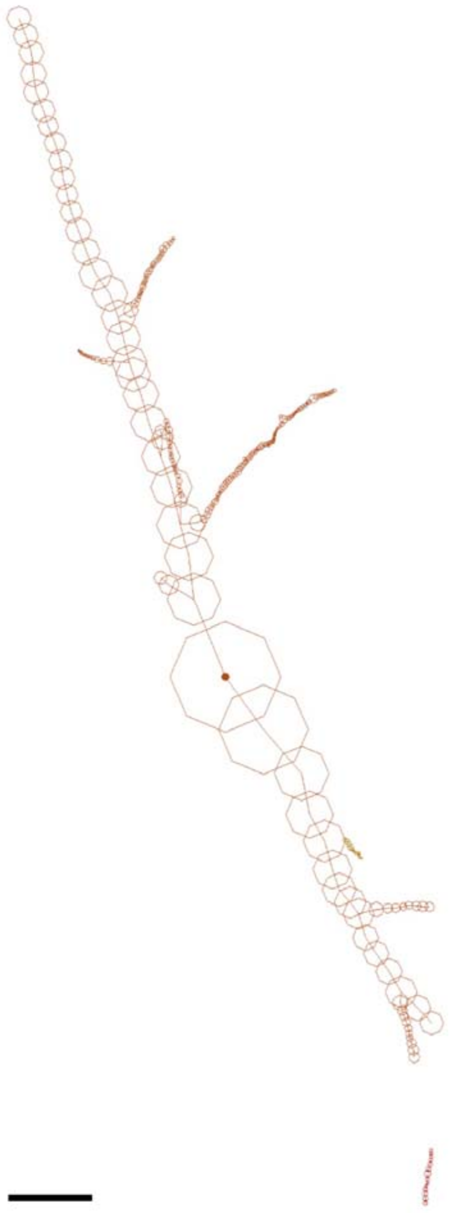

(**I**) N6E structure.

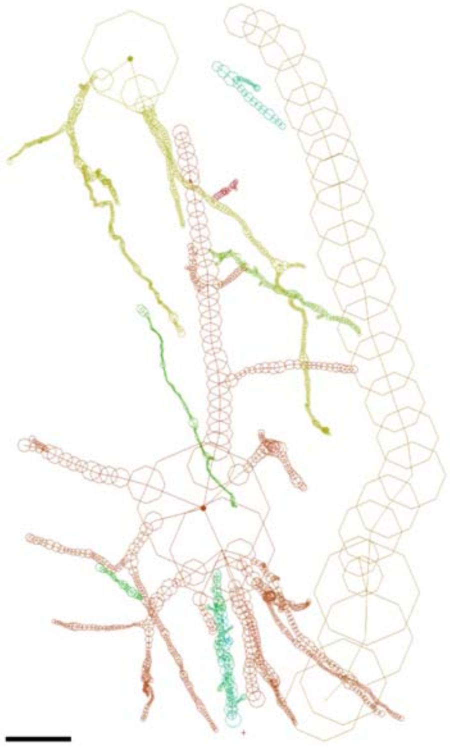

(**J**) N6F structure.

S3 Fig (cont'd). Cartesian coordinate models of control case structures. The pial surface is toward the top. The models were drawn with the MCTrace software. Constituents of the models are color-coded. Nodes composing each constituent are indicated with octagons. Dots indicate somata nodes. Scale bars: 10 μm.

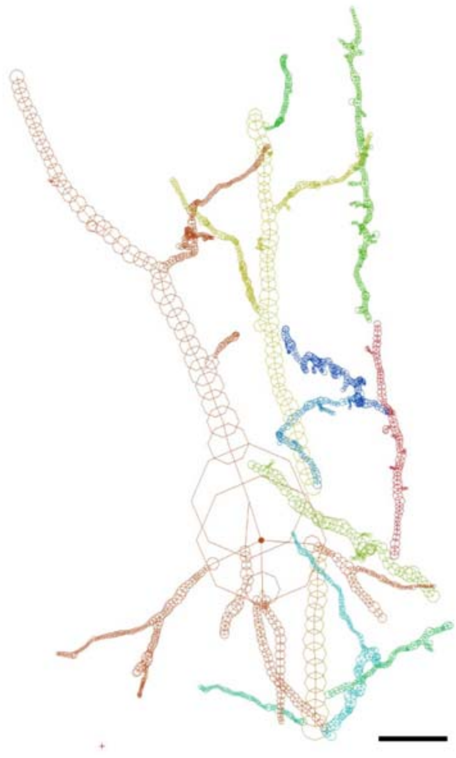

(**K**) N6G structure.

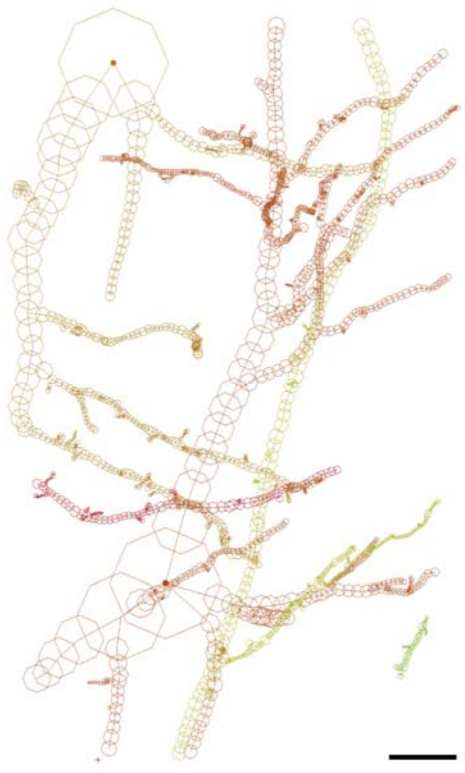

(**L**) N6H structure.

S3 Fig (cont'd). Cartesian coordinate models of control case structures. The pial surface is toward the top. The models were drawn with the MCTrace software. Constituents of the models are color-coded. Nodes composing each constituent are indicated with octagons. Dots indicate somata nodes. Scale bars: 10 μm.

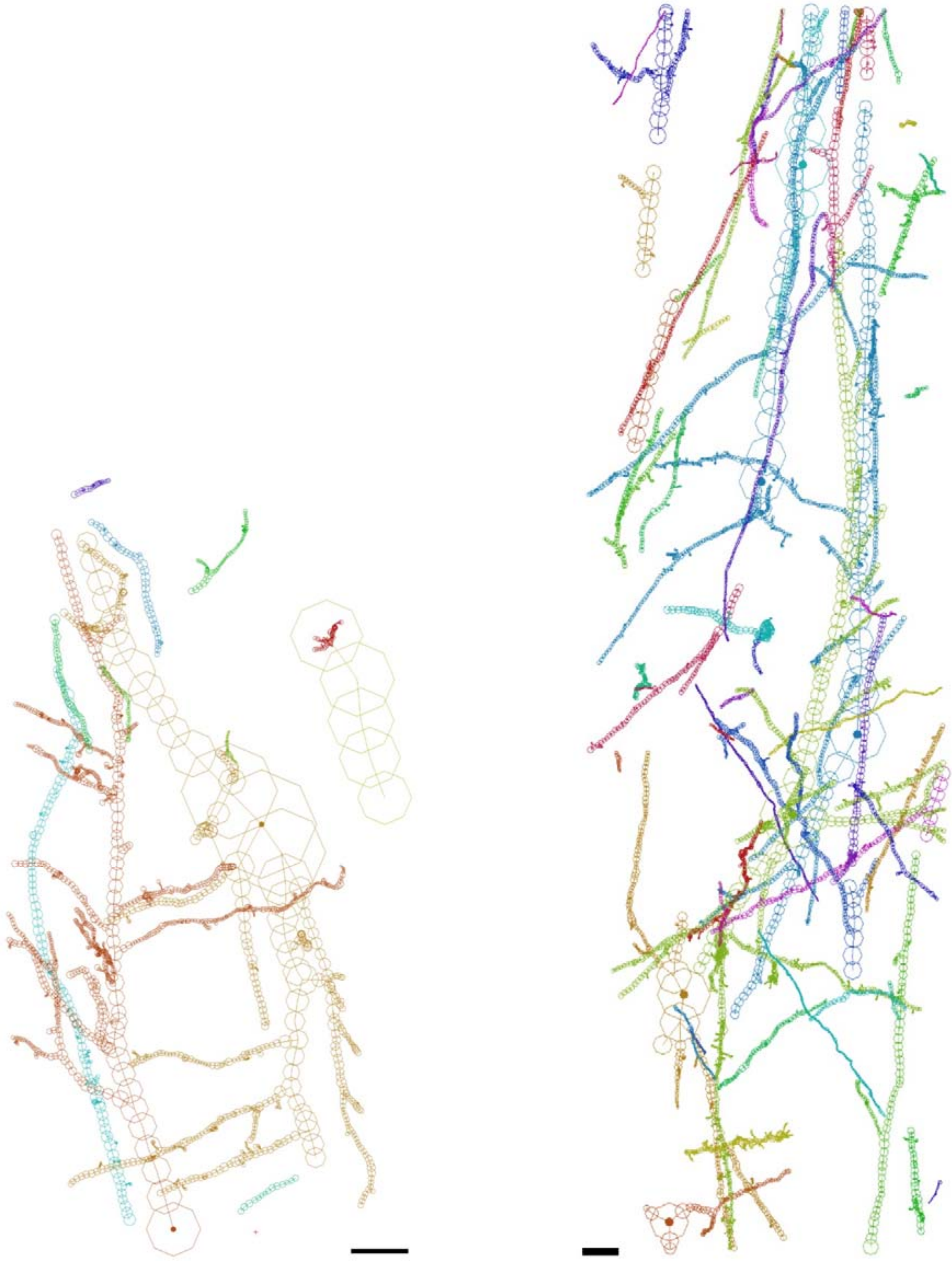

(**M**) N6I structure. (**N**) N7B structure.

S3 Fig (cont'd). Cartesian coordinate models of control case structures. The pial surface is toward the top. The models were drawn with the MCTrace software. Constituents of the models are color-coded. Nodes composing each constituent are indicated with octagons. Dots indicate somata nodes. Scale bars: 10 μm.

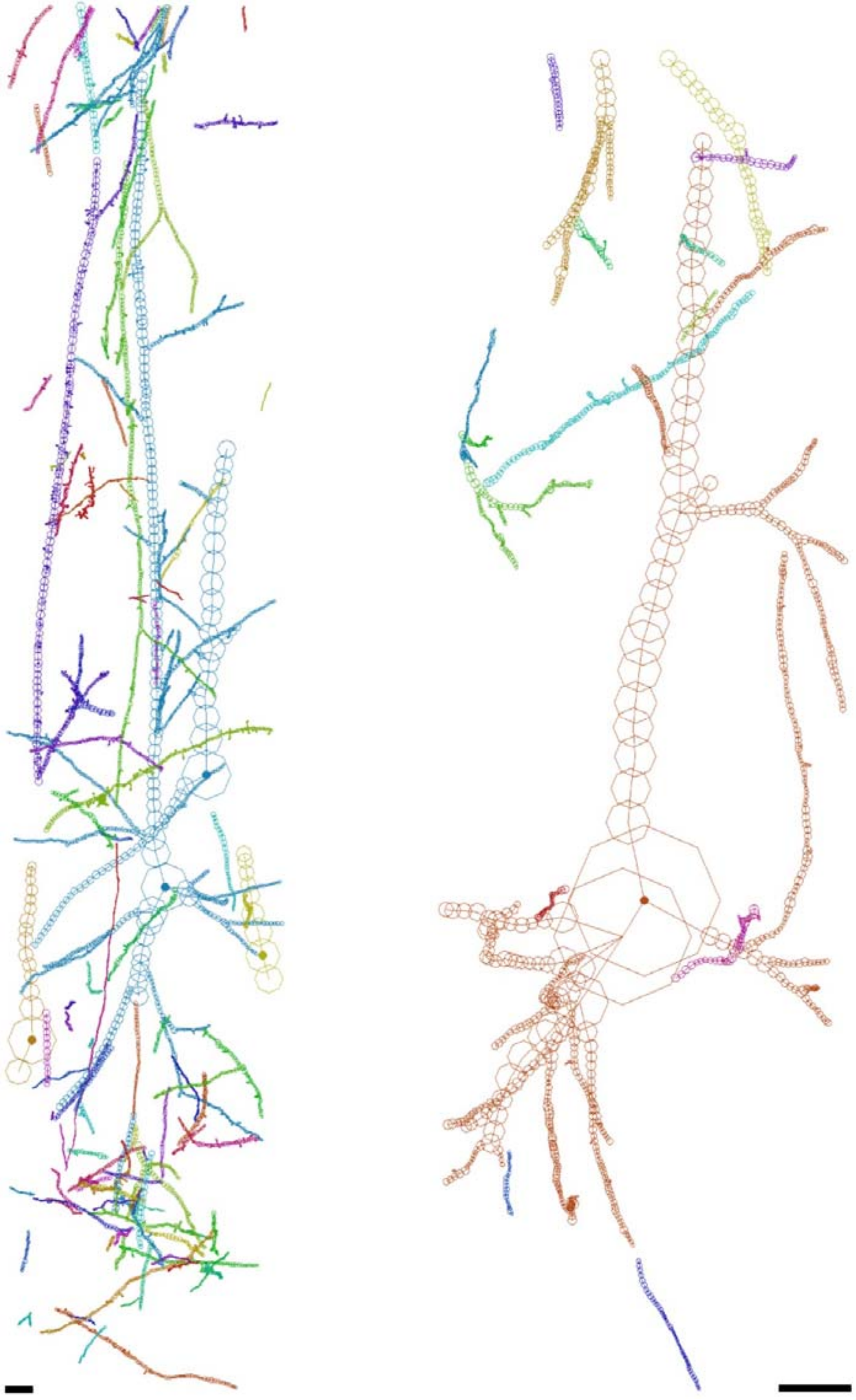

(**O**) N7C structure.

(**P**) N7D structure.

S3 Fig (cont'd). Cartesian coordinate models of control case structures. The pial surface is toward the top. The models were drawn with the MCTrace software. Constituents of the models are color-coded. Nodes composing each constituent are indicated with octagons. Dots indicate somata nodes. Scale bars: 10 μm.

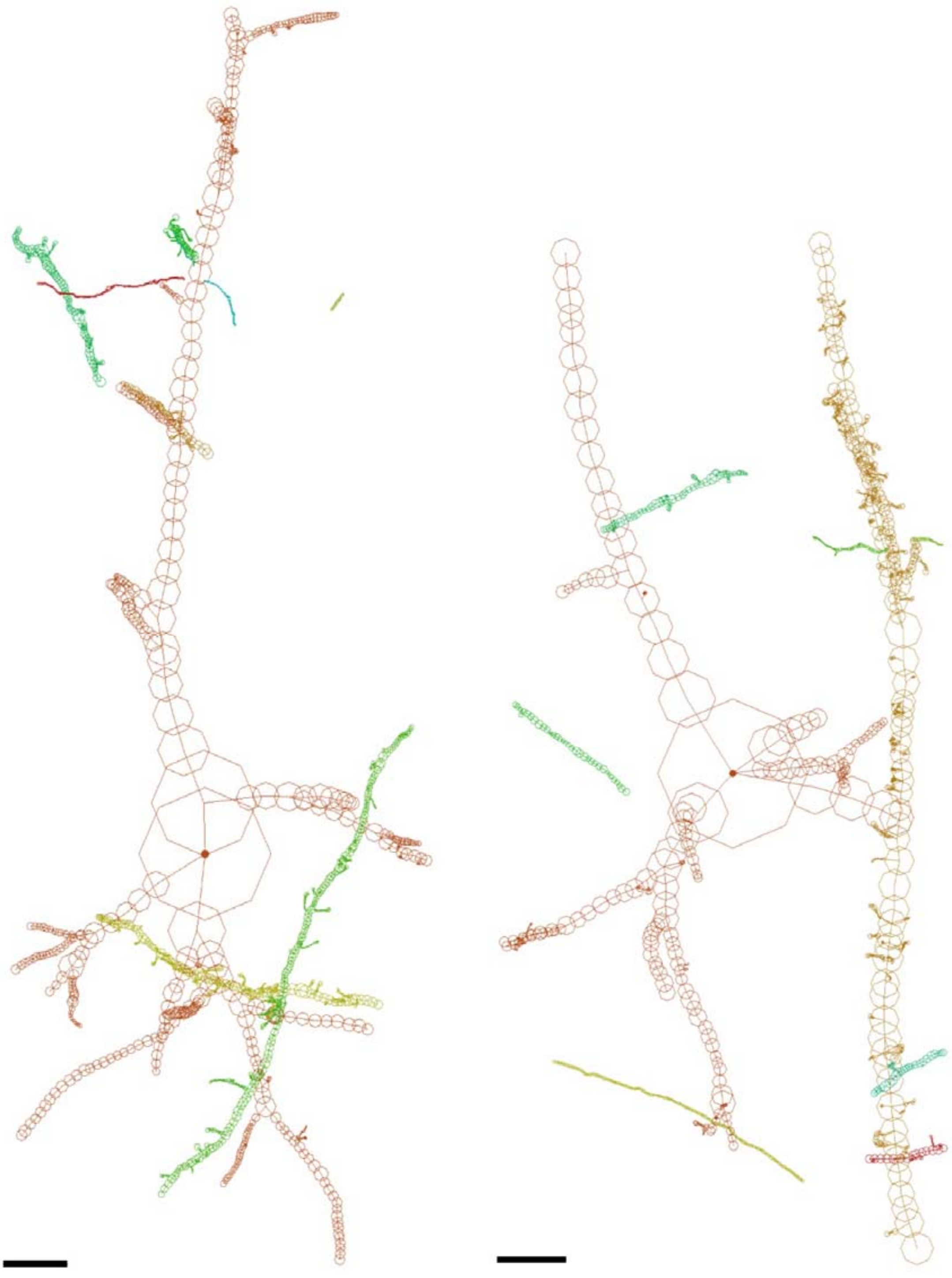

(**Q**) N7E structure. (**R**) N7F structure.

S3 Fig (cont'd). Cartesian coordinate models of control case structures. The pial surface is toward the top. The models were drawn with the MCTrace software. Constituents of the models are color-coded. Nodes composing each constituent are indicated with octagons. Dots indicate somata nodes. Scale bars: 10 μm.

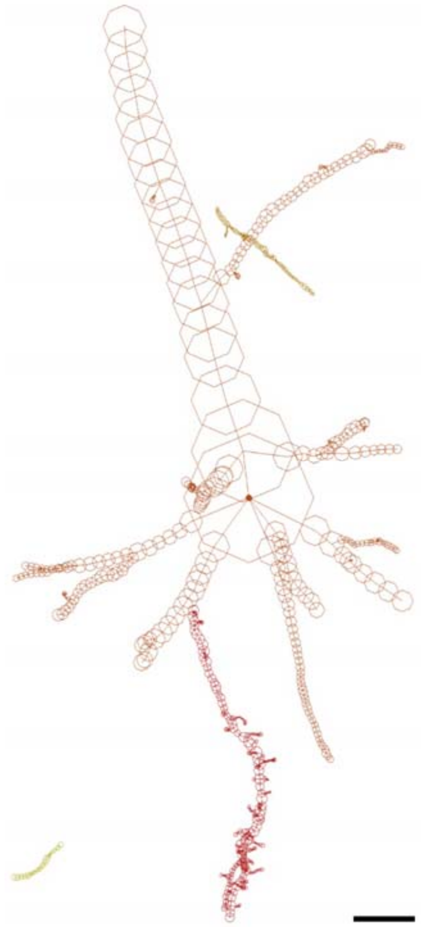

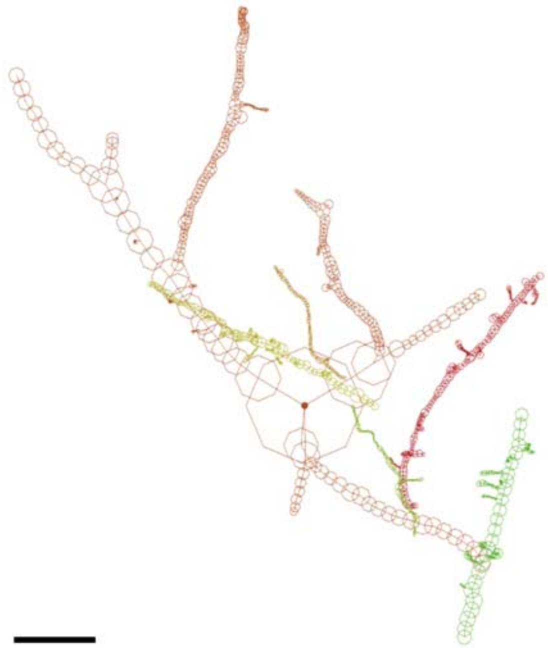

(**S**) N7G structure.

(**T**) N7H structure.

S3 Fig (cont'd). Cartesian coordinate models of control case structures. The pial surface is toward the top. The models were drawn with the MCTrace software. Constituents of the models are color-coded. Nodes composing each constituent are indicated with octagons. Dots indicate somata nodes. Scale bars: 10 μm.

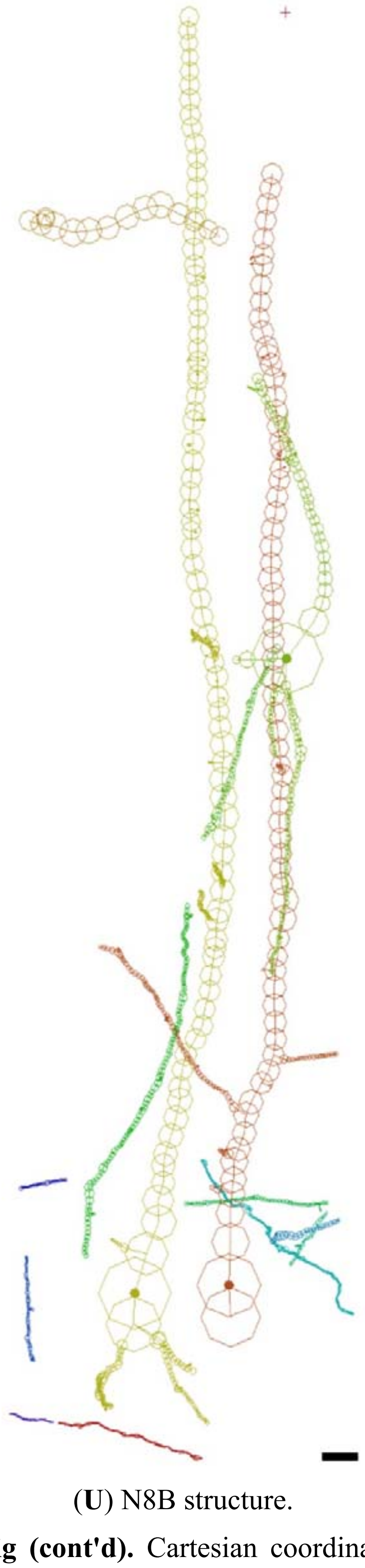

(**U**) N8B structure.

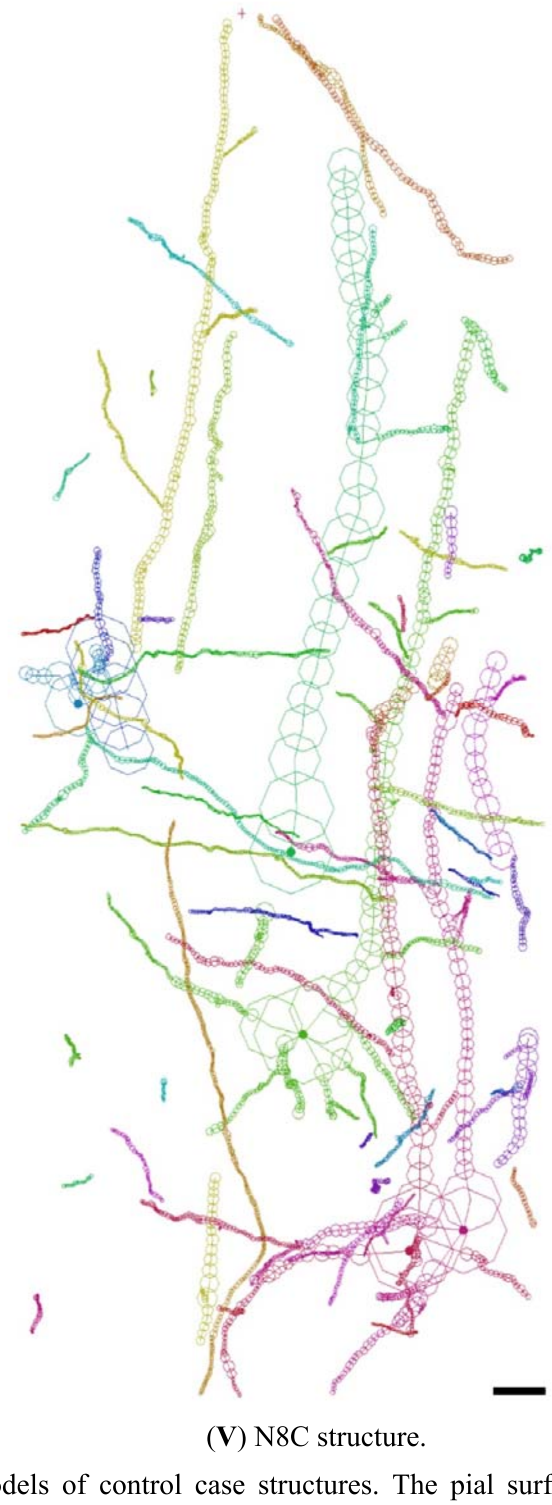

(**V**) N8C structure.

S3 Fig (cont'd). Cartesian coordinate models of control case structures. The pial surface is toward the top. The models were drawn with the MCTrace software. Constituents of the models are color-coded. Nodes composing each constituent are indicated with octagons. Dots indicate somata nodes. Scale bars: 10 μm.

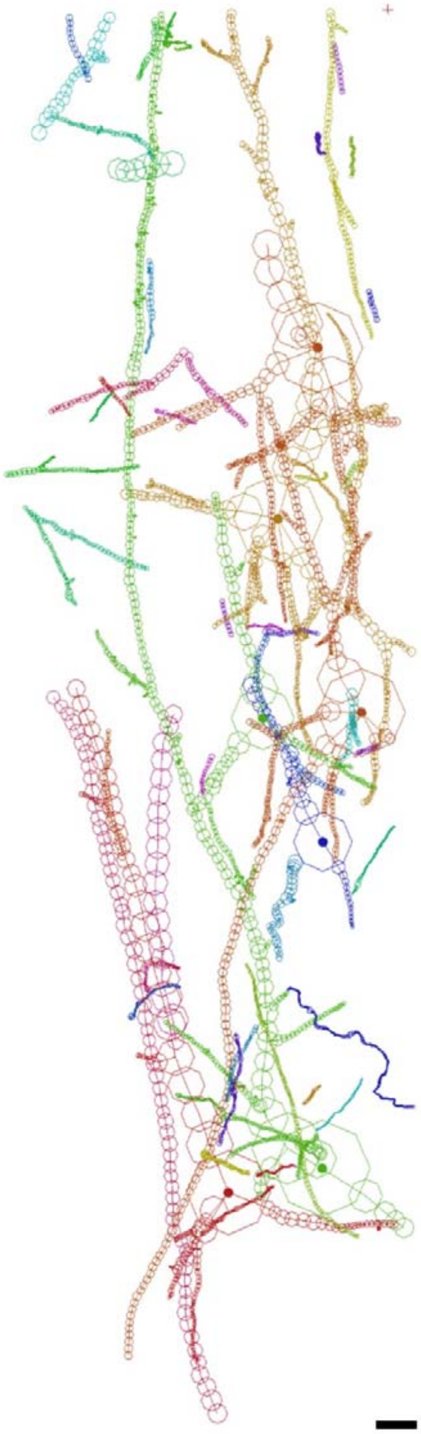

(**W**) N8D structure.

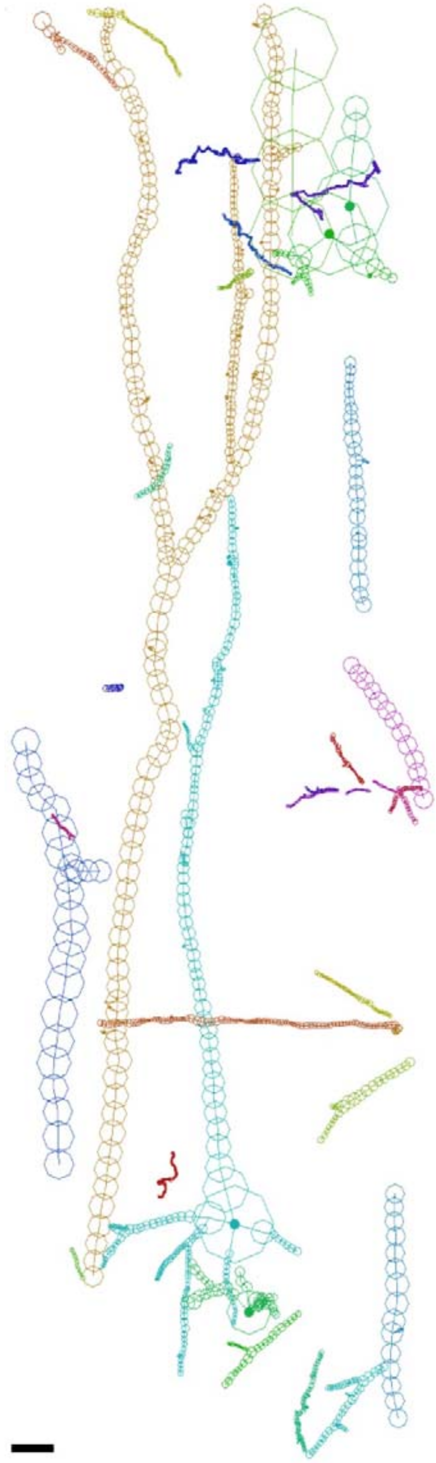

(**X**) N8E structure.

S3 Fig (cont'd). Cartesian coordinate models of control case structures. The pial surface is toward the top. The models were drawn with the MCTrace software. Constituents of the models are color-coded. Nodes composing each constituent are indicated with octagons. Dots indicate somata nodes. Scale bars: 10 µm.

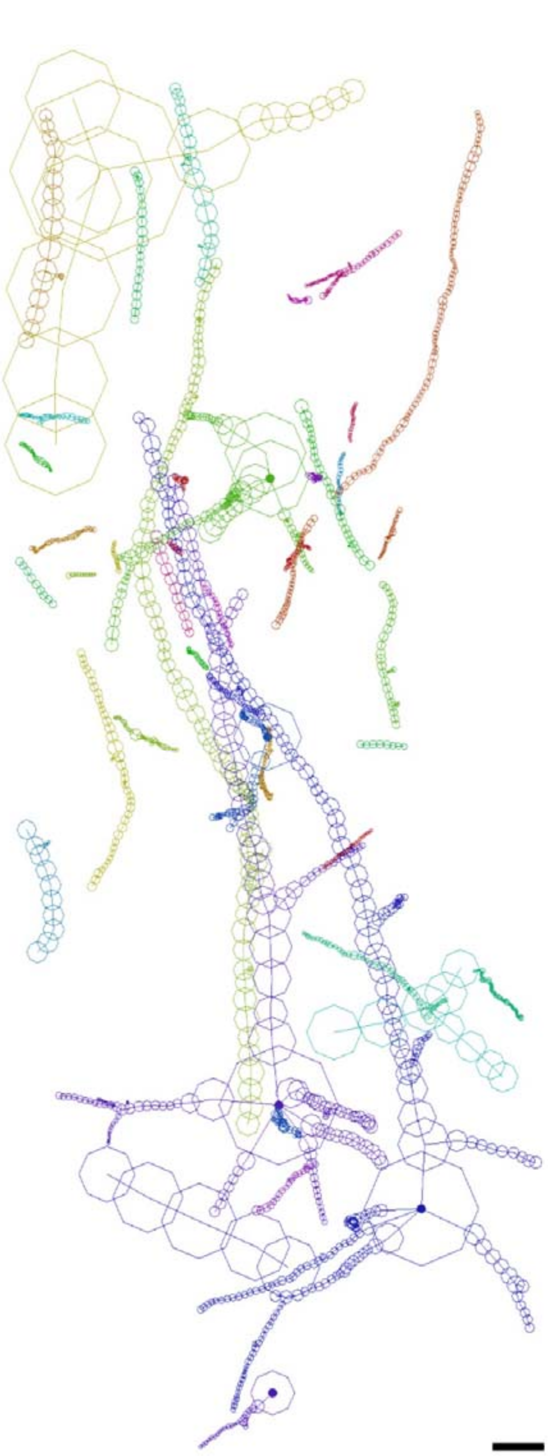

(**Y**) N8F structure.

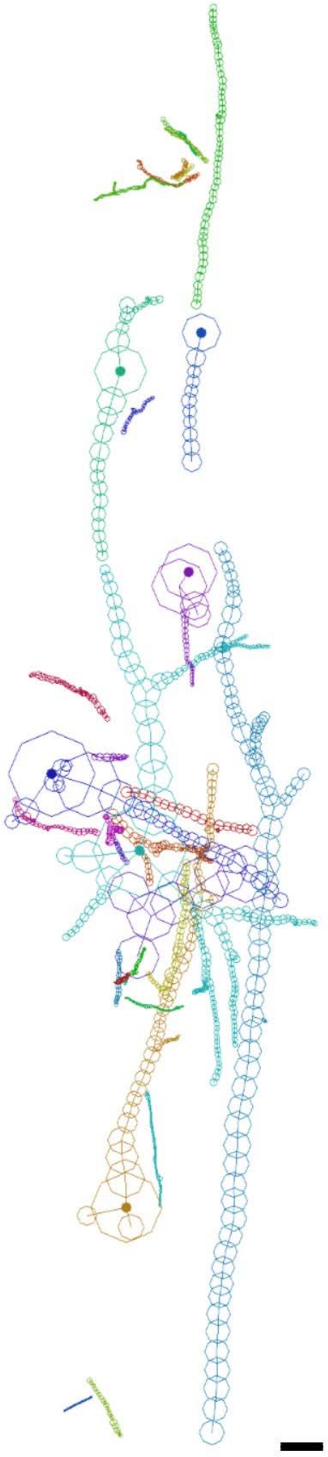

(**Z**) N8G structure.

S3 Fig (cont'd). Cartesian coordinate models of control case structures. The pial surface is toward the top. The models were drawn with the MCTrace software. Constituents of the models are color-coded. Nodes composing each constituent are indicated with octagons. Dots indicate somata nodes. Scale bars: 10 µm.
