# Supplementary material for: Structural aging of human neurons is opposite of the changes in schizophrenia
Source: PLoS One. 2023 Jun 23;18(6):e0287646. doi: 10.1371/journal.pone.0287646 (PMC10289376; doi:10.1371/journal.pone.0287646)
Supplement: S4 Fig — Layer V is indicated with a box and is colored red. (A) Overall structure of the S6 sample. Linear attenuation coefficients of 25–100 cm-1 were rendered with the scatter HQ algorithm using the VG Studio software. Image height: 1900 μm. (B) Overall structure of the N5 sample. Linear attenuation coefficients of 10–100 cm-1 are rendered. Image height: 3520 μm. (PDF) [file pone.0287646.s004.pdf]

A **B**

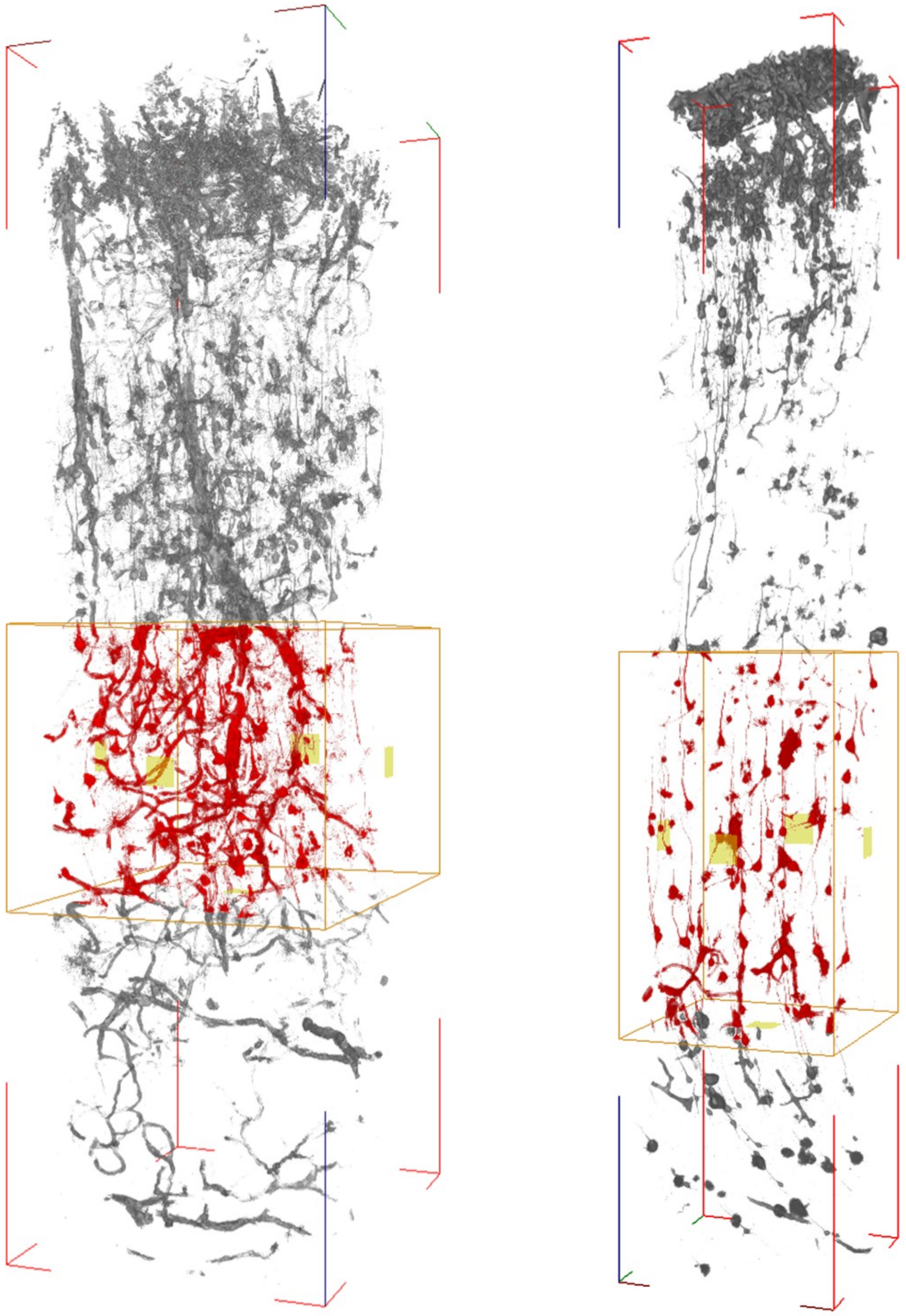

S4 Fig. Overall three-dimensional structure of S6 and N5 samples. Layer V is indicated with a box and is colored red. (**A**) Overall structure of the S6 sample. Linear attenuation coefficients of 25–100 cm^{-1} were rendered with the scatter HQ algorithm using the VG Studio software. Image height: 1900 µm. (**B**) Overall structure of the N5 sample. Linear attenuation coefficients of 10–100 cm^{-1} are rendered. Image height: 3520 µm.
