# Supplementary material for: Structural aging of human neurons is opposite of the changes in schizophrenia
Source: PLoS One. 2023 Jun 23;18(6):e0287646. doi: 10.1371/journal.pone.0287646 (PMC10289376; doi:10.1371/journal.pone.0287646)
Supplement: S5 Fig — Schizophrenia cases are plotted with circles and controls with triangles. (PDF) [file pone.0287646.s005.pdf]

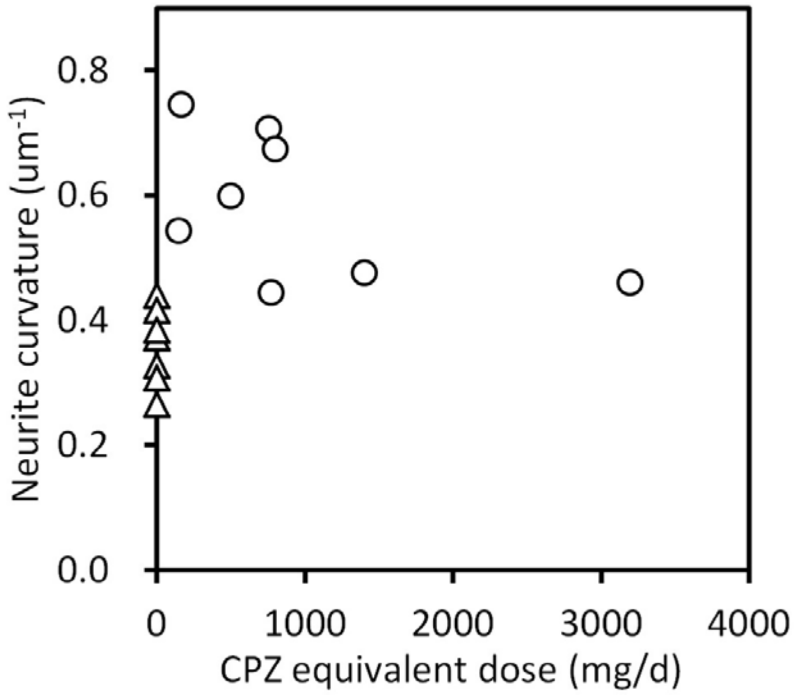

S5 Fig. Scatter plot of neurite curvature and chlorpromazine equivalent dose. Schizophrenia cases are plotted with circles and controls with triangles.
