# Supplementary material for: Structural aging of human neurons is opposite of the changes in schizophrenia
Source: PLoS One. 2023 Jun 23;18(6):e0287646. doi: 10.1371/journal.pone.0287646 (PMC10289376; doi:10.1371/journal.pone.0287646)
Supplement: S1 Table — (PDF) [file pone.0287646.s006.pdf]

S1 Table. Statistics of structural analysis.

Case code	S5	S6	S7	S8	N5	N6	N7	N8
Gender	female	female	female	male	female	male	female	male
Age	62	63	61	59	47	45	42	66
Postmortem interval (hour)	4	2	12	6.5	11	44	28.5	3
Psychiatric record	schizophrenia	schizophrenia	schizophrenia	schizophrenia	no	no	no	no
Tissue structure								
Layer V depth (um)	850-1400	800-1250	1300-1900	1400-2500	1650-2750	1350-2050	2150-3000	1050-1750
Number of datasets	9	5	5	5	6	9	8	7
Number of model nodes	31409	53466	36010	42672	29613	29361	35862	16776
Total length (um) [1]	13616.5	21220.6	20241.1	15038.3	17402.2	19722.9	23336.4	18874.9
Pyramidal process (um)	5390.6	4091.9	8105.6	6474.1	12093.4	10362.3	8435.8	8559.7
Interneuron process (um)	0.0	0.0	38.1	0.0	0.0	66.2	0.0	387.7
Non-typed neuron process (um)	244.4	251.4	0.0	797.0	0.0	1044.5	259.5	527.2
Orphan neurite (um)	6401.2	10574.7	11997.5	7628.6	4756.8	7624.5	13930.4	8372.7
Gliaform cell process (um)	934.5	5162.5	0.0	0.0	0.0	0.0	710.7	78.6
Blood capillary (um)	646.3	1140.2	99.8	138.7	552.0	625.3	0.0	949.2
Number of neurite segments	362	534	715	483	532	570	687	554
Neurite curvature (um^{-1}) [2]	0.54 (0.31)	0.67 (0.46)	0.44 (0.32)	0.74 (0.42)	0.37 (0.23)	0.38 (0.25)	0.31 (0.24)	0.26 (0.19)
Neurite radius (um) [2]	0.54 (0.61)	0.43 (0.49)	0.58 (0.55)	0.42 (0.55)	0.56 (0.49)	0.64 (0.56)	0.77 (0.70)	1.02 (1.02)
Number of spines	2345	3428	1069	2620	1956	959	2371	388
Spine curvature (um^{-1}) [2]	1.40 (0.61)	1.43 (0.61)	1.32 (0.64)	1.42 (0.68)	1.40 (0.65)	1.26 (0.65)	1.23 (0.59)	1.01 (0.66)
Spine radius (um) [2]	0.21 (0.06)	0.19 (0.06)	0.22 (0.07)	0.22 (0.06)	0.19 (0.06)	0.22 (0.08)	0.23 (0.08)	0.27 (0.08)
Spine length (um) [2]	1.50 (0.79)	1.38 (0.78)	1.08 (0.58)	1.23 (0.71)	1.12 (0.65)	1.00 (0.62)	1.29 (0.74)	0.91 (0.48)
Spine density (um^{-1}) [3]	0.238	0.297	0.092	0.224	0.143	0.076	0.145	0.044

[1] Spine length is not included.

[2] Mean (standard deviation)

[3] Spine density = number of spines / total length of spiny dendrite
