# Supplementary material for: Structural aging of human neurons is opposite of the changes in schizophrenia
Source: PLoS One. 2023 Jun 23;18(6):e0287646. doi: 10.1371/journal.pone.0287646 (PMC10289376; doi:10.1371/journal.pone.0287646)
Supplement: S2 Table — (PDF) [file pone.0287646.s007.pdf]

S2 Table. Structural analysis summary.

Case code	S1	S2	S3	S4	S5	S6	S7	S8
Gender	female	female	male	male	female	female	female	male
Age	56	70	64	69	62	63	61	59
Cause of death	sudden death (bronchial obstruction?)	confluent pneumonia	pneumonia, hypoglycemia, decubitus ulcer, cellulitis	lung carcinoma	sudden death, bronchial asthma	asphyxia	sigmoid colon perforation, acute suppurative peritonitis, septic shock	brainstem infarction
Schizophrenia	yes	yes	yes	yes	yes	yes	yes	yes
Onset age	26	31	21	20	50	45	18	35
Duration (year)	30	39	42	49	12	18	43	24
CPZ[1] equivalent dose (mg/d)	3200	1402	500	758	150	800	775	167
Hallucination score	1	1	1	2	0	2	0	1
BA24 structure								
Neurite curvature (um^{-1}) [2]	0.46 (0.28)	0.47 (0.32)	0.60 (0.34)	0.71 (0.36)	0.54 (0.31)	0.67 (0.46)	0.44 (0.32)	0.74 (0.42)
Neurite radius (um) [2]	0.66 (0.61)	0.63 (0.65)	0.58 (0.71)	0.39 (0.34)	0.54 (0.61)	0.43 (0.49)	0.58 (0.55)	0.42 (0.55)
Spine curvature (um^{-1}) [2]	1.13 (0.50)	1.15 (0.52)	1.19 (0.57)	1.16 (0.49)	1.40 (0.61)	1.43 (0.61)	1.32 (0.64)	1.42 (0.68)
Spine radius (um) [2]	0.23 (0.07)	0.21 (0.07)	0.22 (0.08)	0.21 (0.06)	0.21 (0.06)	0.19 (0.06)	0.22 (0.07)	0.22 (0.06)
Spine length (um) [2]	1.62 (0.82)	1.16 (0.70)	1.07 (0.69)	1.32 (0.92)	1.50 (0.79)	1.38 (0.78)	1.08 (0.58)	1.23 (0.71)
Spine density (um^{-1}) [3]	0.422	0.171	0.136	0.261	0.238	0.297	0.092	0.224

[1] Chlorpromazine

[2] Mean (standard deviation)

[3] Spine density = number of spines / total length of spiny dendrite

S2 Table. Structural analysis summary (cont'd).

Case code	N1	N2	N3	N4	N5	N6	N7	N8
Gender	female	female	male	male	female	male	female	male
Age	58	72	62	65	47	45	42	66
Cause of death	traffic injury, blood loss	chronic lymphocytic leukemia	acute myeloid leukemia	primary amyloidosis	subacute brainstem infarction	left middle cerebral artery infarction, brainstem hemorrhage	sudden death (unknown cause)	pancreatic head carcinoma, perforation peritonitis
Schizophrenia	no	no	no	no	no	no	no	no
Onset age								
Duration (year)								
CPZ[1] equivalent dose (mg/d)								
Hallucination score	0	0	0	0	0	0	0	0
BA24 structure								
Neurite curvature (um^{-1}) [2]	0.33 (0.22)	0.44 (0.21)	0.37 (0.21)	0.41 (0.23)	0.37 (0.23)	0.38 (0.25)	0.31 (0.24)	0.26 (0.19)
Neurite radius (um) [2]	1.06 (1.07)	0.53 (0.42)	0.71 (0.73)	0.59 (0.51)	0.56 (0.49)	0.64 (0.56)	0.77 (0.70)	1.02 (1.02)
Spine curvature (um^{-1}) [2]	0.80 (0.54)	1.18 (0.51)	1.16 (0.51)	1.12 (0.53)	1.40 (0.65)	1.26 (0.65)	1.23 (0.59)	1.01 (0.66)
Spine radius (um) [2]	0.29 (0.10)	0.22 (0.06)	0.20 (0.06)	0.22 (0.07)	0.19 (0.06)	0.22 (0.08)	0.23 (0.08)	0.27 (0.08)
Spine length (um) [2]	0.85 (0.80)	1.30 (0.84)	1.36 (0.77)	1.01 (0.60)	1.12 (0.65)	1.00 (0.62)	1.29 (0.74)	0.91 (0.48)
Spine density (um^{-1}) [3]	0.078	0.323	0.247	0.132	0.143	0.076	0.145	0.044

[1] Chlorpromazine

[2] Mean (standard deviation)

[3] Spine density = number of spines / total length of spiny dendrite
