# Supplementary material for: Structural aging of human neurons is opposite of the changes in schizophrenia
Source: PLoS One. 2023 Jun 23;18(6):e0287646. doi: 10.1371/journal.pone.0287646 (PMC10289376; doi:10.1371/journal.pone.0287646)
Supplement: S3 Table — (PDF) [file pone.0287646.s008.pdf]

S3 Table. Conditions of microtomography and nanotomography experiments.

Beamtime start date	2013.10.3	2014.5.27	2019.6.7	2019.10.15	2021.6.1	2011.12.8<br>2012.7.8	2013.1.23<br>-2020.11.6
Facility	SPring-8	SPring-8	APS	SPring-8	SPring-8	SPring-8	SPring-8
Beamline	BL47XU	BL37XU	32-ID	BL37XU	BL37XU	BL20XU [1]	BL20XU [1]
X-ray energy (keV)	8.0	8.0	8.0	8.0	8.0	12.0	12.0
Contrast	Zernike	Absorption	Absorption	Absorption	Absorption	Absorption	Absorption
Beam condenser	Sector zone plate	Sector zone plate	CRL[2] + capillary	Sector zone plate	Sector zone plate	-	-
Outermost zone width (nm) / diameter (um) of Fresnel zone plate	100 / 155	100 / 310	50 / 180	100 / 310	100 / 310	-	-
Focal depth (um) [3]	258	258	65	258	258	-	-
Scintillator screen	P43<br>(Gd_2O_2S:Tb)	P43	LuAG:Ce<br>($Lu_3Al_5O_{12}$:Ce)	P43	P43	LSO<br>(Lu_2SiO_5:Ce)	LSO, LuAG:Ce<br>or GAGG:Ce<br>($Gd_3Al_2Ga_3O_{12}$:Ce)
Pixel size (nm)	40.2	59.6	22.5	48.7	48.1	500	500
X-ray illumination (diameter in pixels)	1670	2400	Full field	1850	Full field	Full field	Full field
Viewing field (pixels) [4]	1680 x 1680	2048 x 2048	2448 x 2048	2048 x 2048	2048 x 2048	1920 x 1440	2048 x 2048
Maximum image width (um)	67	122	55	100	99	960	1024
Image dynamic range (bits) [5]	16	16	13	14	14	12	14-16
Number of sample frames per dataset	900	1800	1815	1800	1800	1800	1800
Degrees per frame	0.200	0.100	0.099	0.100	0.100	0.100	0.100
Exposure time per frame (msec)	500	700	500	200	200	150-200	80-200
Data collection time (sec)	600	2400	1200	720	800	800-1200	250-600
Spatial resolution (nm)	180 [6]- 220 [7]	200 [6]- 250 [7]	200 [6,7]	200 [6] - 250 [7]	200 [6] - 250 [7]	1200 [6]	1200 [6]

[1] Used for visualizing overall sample structures

[2] CRL: compound refractive lens

[3] Focal depth Δf was calculated with $\Delta f = \pm \lambda / (2\ NA^2)$ and $NA = \lambda / (2\Delta r_N)$,
where λ is the wavelength, NA is the numerical aperture, and Δr_N is the outermost zone width of Fresnel zone plate.

[4] Width x height

[5] Defined from the maximum intensity of flat field images

[6] Determined using three-dimensional square-wave test patterns. These estimates represent the resolution that the instruments can reach.

[7] Determined from the Fourier domain plot. These estimates represent the resolution of the sample image itself.
