# Supplementary material for: Structural aging of human neurons is opposite of the changes in schizophrenia
Source: PLoS One. 2023 Jun 23;18(6):e0287646. doi: 10.1371/journal.pone.0287646 (PMC10289376; doi:10.1371/journal.pone.0287646)
Supplement: S4 Table — (A–D) Schizophrenia cases. (E–H) Control cases. (PDF) [file pone.0287646.s009.pdf]

S4 Table. Statistics of datasets and Cartesian coordinate models. (**A**) Schizophrenia case S5.

Dataset name	S5A	S5B	S5C	S5D	S5E
Beamtime start date	2021.6.1	2021.6.1	2021.6.1	2021.6.1	2021.6.1
Image size (voxel) [1]	2060 x 2090 x 7075	2040 x 2040 x 3717	2050 x 2080 x 3715	2070 x 2080 x 3719	2070 x 2070 x 5372
Image size (um) [1]	99.1 x 100.5 x 340.3	98.1 x 98.1 x 178.8	98.6 x 100.0 x 178.7	99.6 x 100.0 x 178.9	99.6 x 99.6 x 258.4
Cortical depth of upper end (um)	800	920	1360	1360	1150
Number of model nodes	10327	1347	3044	2572	2335
Number of constituents	35	8	22	23	14
Pyramidal neurons	2	2	1	1	1
Interneurons	0	0	0	0	0
Non-typed neurons	0	0	0	0	0
Orphan neurites	32	1	21	21	11
Gliaform cells	1	0	0	0	0
Blood capillaries	0	5	0	1	2
Total length (um) [2]	3523.0	861.3	1216.9	1309.1	1326.6
Pyramidal process (um)	915.3	367.4	422.3	473.5	894.3
Interneuron process (um)	0.0	0.0	0.0	0.0	0.0
Non-typed neuron process (um)	0.0	0.0	0.0	0.0	0.0
Orphan neurite (um)	1673.2	171.4	794.6	771.6	349.0
Gliaform cell process (um)	934.5	0.0	0.0	0.0	0.0
Blood capillary (um)	0.0	322.6	0.0	64.1	83.4
Number of neurite segments	75	17	40	34	30
Neurite curvature (um^{-1}) [3]	0.60 (0.34)	0.52 (0.39)	0.58 (0.32)	0.45 (0.30)	0.49 (0.25)
Neurite radius (um) [3]	0.57 (0.66)	0.57 (0.45)	0.50 (0.43)	0.53 (0.39)	0.44 (0.20)
Number of spines	1014	77	243	150	97
Spine curvature (um^{-1}) [3]	1.39 (0.56)	1.72 (0.76)	1.48 (0.74)	1.18 (0.51)	1.40 (0.69)
Spine radius (um) [3]	0.19 (0.05)	0.19 (0.06)	0.22 (0.06)	0.24 (0.07)	0.23 (0.07)
Spine length (um) [3]	1.60 (0.78)	1.30 (0.77)	1.23 (0.68)	1.59 (0.69)	1.41 (0.90)
Spine density (um^{-1}) [4]	0.432	0.165	0.261	0.142	0.097

[1] Image width x height x number of slices

[2] Spine length is not included.

[3] Mean (standard deviation)

[4] Spine density = number of spines / total length of spiny dendrite

S4 Table. Statistics of datasets and Cartesian coordinate models. (**A**) Schizophrenia case S5 (cont'd).

Dataset name	S5F	S5G	S5H	S5I
Beamtime start date	2021.6.1	2021.6.1	2021.6.1	2021.6.1
Image size (voxel) [1]	2090 x 2020 x 3698	2060 x 2040 x 3712	2030 x 2030 x 2048	2020 x 2020 x 2048
Image size (um) [1]	100.5 x 97.2 x 177.9	99.1 x 98.1 x 178.5	97.6 x 97.6 x 98.5	97.2 x 97.2 x 98.5
Cortical depth of upper end (um)	1180	980	1060	1030
Number of model nodes	2379	3050	2987	3368
Number of constituents	32	20	28	17
Pyramidal neurons	1	1	1	1
Interneurons	0	0	0	0
Non-typed neurons	0	1	0	0
Orphan neurites	30	17	27	16
Gliaform cells	0	0	0	0
Blood capillaries	1	1	0	0
Total length (um) [2]	1300.4	1480.2	1127.8	1471.2
Pyramidal process (um)	460.7	522.6	454.7	879.8
Interneuron process (um)	0.0	0.0	0.0	0.0
Non-typed neuron process (um)	0.0	244.4	0.0	0.0
Orphan neurite (um)	755.4	621.4	673.1	591.5
Gliaform cell process (um)	0.0	0.0	0.0	0.0
Blood capillary (um)	84.3	91.9	0.0	0.0
Number of neurite segments	43	37	46	40
Neurite curvature (um^{-1}) [3]	0.55 (0.30)	0.51 (0.24)	0.57 (0.30)	0.50 (0.28)
Neurite radius (um) [3]	0.60 (1.11)	0.57 (0.61)	0.48 (0.37)	0.54 (0.56)
Number of spines	132	192	214	226
Spine curvature (um^{-1}) [3]	1.34 (0.55)	1.33 (0.57)	1.48 (0.59)	1.39 (0.67)
Spine radius (um) [3]	0.23 (0.07)	0.20 (0.05)	0.20 (0.06)	0.21 (0.07)
Spine length (um) [3]	1.28 (0.53)	1.70 (0.84)	1.53 (0.90)	1.36 (0.79)
Spine density (um^{-1}) [4]	0.164	0.150	0.278	0.188

[1] Image width x height x number of slices

[2] Spine length is not included.

[3] Mean (standard deviation)

[4] Spine density = number of spines / total length of spiny dendrite

S4 Table. Statistics of datasets and Cartesian coordinate models. (**B**) Schizophrenia case S6.

Dataset name	S6A	S6B	S6C	S6D	S6E
Beamtime start date	2019.10.15	2019.10.15	2019.10.15	2019.6.7	2019.6.7
Image size (voxel) [1]	2050 x 2060 x 6998	2030 x 2030 x 3692	2050 x 2080 x 5348	1220 x 1220 x 4307	1220 x 1210 x 3485
Image size (um) [1]	99.8 x 100.3 x 340.8	98.9 x 98.9 x 179.8	99.8 x 101.3 x 260.4	54.9 x 54.9 x 193.8	54.9 x 54.5 x 156.8
Cortical depth of upper end (um)	810	1090	720	880	750
Number of model nodes	16856	7474	20416	4945	3775
Number of constituents	112	89	84	35	33
Pyramidal neurons	1	1	2	1	1
Interneurons	0	0	0	0	0
Non-typed neurons	0	0	1	0	1
Orphan neurites	106	85	76	32	29
Gliaform cells	0	0	2	0	1
Blood capillaries	5	3	3	2	1
Total length (um) [2]	5183.7	3011.0	9698.6	1522.5	1804.8
Pyramidal process (um)	1370.5	623.5	1548.8	392.7	156.4
Interneuron process (um)	0.0	0.0	0.0	0.0	0.0
Non-typed neuron process (um)	0.0	0.0	118.8	0.0	132.6
Orphan neurite (um)	3618.9	1949.9	3444.3	914.4	647.2
Gliaform cell process (um)	0.0	0.0	4351.3	0.0	811.2
Blood capillary (um)	194.3	437.6	235.4	215.4	57.5
Number of neurite segments	168	116	156	51	43
Neurite curvature (um^{-1}) [3]	0.62 (0.41)	0.89 (0.51)	0.58 (0.40)	0.79 (0.55)	0.51 (0.32)
Neurite radius (um) [3]	0.41 (0.31)	0.37 (0.57)	0.48 (0.52)	0.34 (0.27)	0.64 (0.79)
Number of spines	1382	302	1283	237	224
Spine curvature (um^{-1}) [3]	1.37 (0.57)	1.50 (0.63)	1.40 (0.61)	1.74 (0.66)	1.63 (0.59)
Spine radius (um) [3]	0.20 (0.05)	0.19 (0.06)	0.20 (0.06)	0.14 (0.05)	0.15 (0.05)
Spine length (um) [3]	1.47 (0.75)	1.26 (0.81)	1.33 (0.77)	1.44 (0.85)	1.28 (0.76)
Spine density (um^{-1}) [4]	0.336	0.169	0.316	0.250	0.349

[1] Image width x height x number of slices

[2] Spine length is not included.

[3] Mean (standard deviation)

[4] Spine density = number of spines / total length of spiny dendrite

S4 Table. Statistics of datasets and Cartesian coordinate models. (**C**) Schizophrenia case S7.

Dataset name	S7A	S7B	S7C	S7D	S7E
Beamtime start date	2014.5.27	2014.5.27	2014.5.27	2019.10.15	2019.10.15
Image size (voxel) [1]	2030 x 2030 x 3225	2040 x 2030 x 3223	2020 x 2020 x 2048	2080 x 2120 x 6989	2040 x 2040 x 5337
Image size (um) [1]	121.0 x 121.0 x 192.2	121.6 x 121.0 x 192.1	120.4 x 120.4 x 122.1	101.3 x 103.2 x 340.4	99.3 x 99.3 x 259.9
Cortical depth of upper end (um)	1480	1300	1400	1500	1200
Number of model nodes	8607	8053	6285	10456	2609
Number of constituents	49	80	65	158	39
Pyramidal neurons	2	2	2	8	4
Interneurons	0	0	0	1	0
Non-typed neurons	0	0	0	0	0
Orphan neurites	46	78	63	149	35
Gliaform cells	0	0	0	0	0
Blood capillaries	1	0	0	0	0
Total length (um) [2]	4379.4	4105.0	3350.3	6779.2	1627.2
Pyramidal process (um)	1032.2	1421.3	1417.0	3427.6	807.5
Interneuron process (um)	0.0	0.0	0.0	38.1	0.0
Non-typed neuron process (um)	0.0	0.0	0.0	0.0	0.0
Orphan neurite (um)	3247.4	2683.7	1933.3	3313.4	819.7
Gliaform cell process (um)	0.0	0.0	0.0	0.0	0.0
Blood capillary (um)	99.8	0.0	0.0	0.0	0.0
Number of neurite segments	96	143	121	271	84
Neurite curvature (um^{-1}) [3]	0.48 (0.28)	0.48 (0.30)	0.47 (0.24)	0.42 (0.36)	0.37 (0.32)
Neurite radius (um) [3]	0.46 (0.30)	0.50 (0.38)	0.58 (0.68)	0.59 (0.46)	0.79 (0.90)
Number of spines	216	257	153	273	170
Spine curvature (um^{-1}) [3]	1.28 (0.61)	1.22 (0.54)	1.41 (0.72)	1.44 (0.67)	1.26 (0.64)
Spine radius (um) [3]	0.22 (0.07)	0.24 (0.07)	0.21 (0.06)	0.21 (0.06)	0.20 (0.06)
Spine length (um) [3]	0.92 (0.56)	1.23 (0.62)	0.94 (0.57)	1.15 (0.57)	1.06 (0.50)
Spine density (um^{-1}) [4]	0.087	0.107	0.073	0.073	0.180

[1] Image width x height x number of slices

[2] Spine length is not included.

[3] Mean (standard deviation)

[4] Spine density = number of spines / total length of spiny dendrite

S4 Table. Statistics of datasets and Cartesian coordinate models. (**D**) Schizophrenia case S8.

Dataset name	S8A	S8B	S8C	S8D	S8E
Beamtime start date	2021.6.1	2021.6.1	2021.6.1	2021.6.1	2021.6.1
Image size (voxel) [1]	2010 x 2000 x 7073	2050 x 2060 x 7068	2070 x 2090 x 8756	2050 x 2040 x 3722	2070 x 2050 x 8762
Image size (um) [1]	96.7 x 96.2 x 340.2	98.6 x 99.1 x 340.0	99.6 x 100.5 x 421.2	98.6 x 98.1 x 179.0	99.6 x 98.6 x 421.5
Cortical depth of upper end (um)	1340	1580	1540	2260	2140
Number of model nodes	6855	9945	7537	6137	12198
Number of constituents	19	91	32	24	110
Pyramidal neurons	1	1	3	0	2
Interneurons	0	0	0	0	0
Non-typed neurons	0	0	0	1	0
Orphan neurites	14	90	29	23	108
Gliaform cells	0	0	0	0	0
Blood capillaries	4	0	0	0	0
Total length (um) [2]	2707.2	3264.4	3053.8	1865.7	4147.2
Pyramidal process (um)	1830.3	873.2	2226.7	0.0	1543.9
Interneuron process (um)	0.0	0.0	0.0	0.0	0.0
Non-typed neuron process (um)	0.0	0.0	0.0	797.0	0.0
Orphan neurite (um)	738.2	2391.2	827.1	1068.7	2603.4
Gliaform cell process (um)	0.0	0.0	0.0	0.0	0.0
Blood capillary (um)	138.7	0.0	0.0	0.0	0.0
Number of neurite segments	59	122	87	52	163
Neurite curvature (um^{-1}) [3]	0.58 (0.37)	0.91 (0.41)	0.61 (0.41)	0.63 (0.42)	0.79 (0.39)
Neurite radius (um) [3]	0.49 (0.32)	0.29 (0.23)	0.65 (0.87)	0.57 (0.99)	0.32 (0.24)
Number of spines	534	246	442	674	724
Spine curvature (um^{-1}) [3]	1.39 (0.65)	1.47 (0.77)	1.38 (0.64)	1.42 (0.64)	1.44 (0.71)
Spine radius (um) [3]	0.23 (0.06)	0.22 (0.07)	0.24 (0.06)	0.20 (0.05)	0.23 (0.06)
Spine length (um) [3]	1.25 (0.73)	1.21 (0.66)	1.28 (0.75)	1.25 (0.69)	1.18 (0.70)
Spine density (um^{-1}) [4]	0.241	0.113	0.176	0.387	0.237

[1] Image width x height x number of slices

[2] Spine length is not included.

[3] Mean (standard deviation)

[4] Spine density = number of spines / total length of spiny dendrite

S4 Table. Statistics of datasets and Cartesian coordinate models. (**E**) Control case N5.

Dataset name	N5A	N5B	N5C	N5D	N5E	N5F
Beamtime start date	2021.6.1	2021.6.1	2021.6.1	2021.6.1	2021.6.1	2021.6.1
Image size (voxel) [1]	2070 x 2050 x 7065	2030 x 2030 x 5387	2020 x 2010 x 10424	2020 x 2020 x 8743	2050 x 2060 x 7062	2070 x 2070 x 8740
Image size (um) [1]	99.6 x 98.6 x 339.8	97.6 x 97.6 x 259.1	97.2 x 96.7 x 501.4	97.2 x 97.2 x 420.5	98.6 x 99.1 x 339.7	99.6 x 99.6 x 420.4
Cortical depth of upper end (um)	2320	1930	1760	2000	2240	2440
Number of model nodes	8224	2381	4969	2719	3018	8302
Number of constituents	40	25	24	10	25	41
Pyramidal neurons	3	1	2	1	1	3
Interneurons	0	0	0	0	0	0
Non-typed neurons	0	0	0	0	0	0
Orphan neurites	35	24	22	8	24	37
Gliaform cells	0	0	0	0	0	0
Blood capillaries	2	0	0	1	0	1
Total length (um) [2]	4653.9	1520.5	2695.6	1936.6	1670.9	4924.7
Pyramidal process (um)	3487.5	885.5	1683.1	1531.8	1058.7	3446.8
Interneuron process (um)	0.0	0.0	0.0	0.0	0.0	0.0
Non-typed neuron process (um)	0.0	0.0	0.0	0.0	0.0	0.0
Orphan neurite (um)	1122.1	635.0	1012.5	228.4	612.2	1146.6
Gliaform cell process (um)	0.0	0.0	0.0	0.0	0.0	0.0
Blood capillary (um)	44.3	0.0	0.0	176.4	0.0	331.3
Number of neurite segments	136	57	74	52	58	155
Neurite curvature (um^{-1}) [3]	0.35 (0.19)	0.35 (0.22)	0.34 (0.25)	0.34 (0.24)	0.39 (0.21)	0.41 (0.25)
Neurite radius (um) [3]	0.50 (0.28)	0.55 (0.41)	0.73 (0.85)	0.56 (0.29)	0.47 (0.24)	0.56 (0.53)
Number of spines	483	131	392	221	208	521
Spine curvature (um^{-1}) [3]	1.39 (0.59)	1.32 (0.66)	1.38 (0.65)	1.34 (0.70)	1.45 (0.67)	1.44 (0.66)
Spine radius (um) [3]	0.18 (0.05)	0.20 (0.05)	0.20 (0.06)	0.21 (0.06)	0.18 (0.05)	0.19 (0.06)
Spine length (um) [3]	1.29 (0.69)	1.04 (0.51)	1.09 (0.66)	0.97 (0.59)	1.09 (0.61)	1.09 (0.64)
Spine density (um^{-1}) [4]	0.133	0.121	0.172	0.147	0.142	0.140

[1] Image width x height x number of slices

[2] Spine length is not included.

[3] Mean (standard deviation)

[4] Spine density = number of spines / total length of spiny dendrite

S4 Table. Statistics of datasets and Cartesian coordinate models. (**F**) Control case N6.

Dataset name	N6A	N6B	N6C	N6D	N6E
Beamtime start date	2019.10.15	2019.10.15	2019.6.7	2019.6.7	2019.6.7
Image size (voxel) [1]	2070 x 2080 x 6981	2100 x 2070 x 6988	1240 x 1240 x 4277	1220 x 1220 x 2646	1230 x 1230 x 3477
Image size (um) [1]	100.8 x 101.3 x 340.0	102.3 x 100.8 x 340.3	55.8 x 55.8 x 192.5	54.9 x 54.9 x 119.1	55.4 x 55.4 x 156.5
Cortical depth of upper end (um)	1210	1190	1920	1940	1680
Number of model nodes	9980	11186	791	519	231
Number of constituents	48	89	7	8	3
Pyramidal neurons	4	4	1	1	1
Interneurons	0	0	0	1	0
Non-typed neurons	2	0	0	0	0
Orphan neurites	42	83	6	6	2
Gliaform cells	0	0	0	0	0
Blood capillaries	0	2	0	0	0
Total length (um) [2]	6804.6	7218.8	448.7	426.9	243.1
Pyramidal process (um)	4042.9	3191.7	106.6	216.3	232.0
Interneuron process (um)	0.0	0.0	0.0	66.2	0.0
Non-typed neuron process (um)	483.7	0.0	0.0	0.0	0.0
Orphan neurite (um)	2278.0	3583.3	342.1	144.4	11.1
Gliaform cell process (um)	0.0	0.0	0.0	0.0	0.0
Blood capillary (um)	0.0	443.8	0.0	0.0	0.0
Number of neurite segments	177	174	18	17	12
Neurite curvature (um^{-1}) [3]	0.33 (0.18)	0.44 (0.29)	0.58 (0.34)	0.41 (0.43)	0.33 (0.31)
Neurite radius (um) [3]	0.64 (0.53)	0.47 (0.42)	0.49 (0.56)	1.06 (1.10)	0.93 (0.76)
Number of spines	326	243	9	9	3
Spine curvature (um^{-1}) [3]	1.28 (0.69)	1.43 (0.70)	1.37 (0.76)	1.18 (0.39)	-
Spine radius (um) [3]	0.22 (0.08)	0.20 (0.07)	0.24 (0.09)	0.22 (0.07)	0.22 (0.02)
Spine length (um) [3]	0.84 (0.51)	0.99 (0.65)	0.63 (0.38)	0.98 (0.51)	0.25 (0.04)
Spine density (um^{-1}) [4]	0.067	0.054	0.048	0.055	0.088

[1] Image width x height x number of slices

[2] Spine length is not included.

[3] Mean (standard deviation)

[4] Spine density = number of spines / total length of spiny dendrite

S4 Table. Statistics of datasets and Cartesian coordinate models. (**F**) Control case N6 (cont'd).

Dataset name	N6F	N6G	N6H	N6I
Beamtime start date	2013.10.3	2013.10.3	2013.10.3	2013.10.3
Image size (voxel) [1]	1680 x 1680 x 2747	1660 x 1660 x 2723	1680 x 1680 x 2760	1690 x 1710 x 3828
Image size (um) [1]	67.5 x 67.5 x 110.4	66.7 x 66.7 x 109.5	67.5 x 67.5 x 111.0	67.9 x 68.7 x 153.9
Cortical depth of upper end (um)	1700	1630	1580	1510
Number of model nodes	1551	1508	1654	1941
Number of constituents	10	12	5	14
Pyramidal neurons	1	1	1	2
Interneurons	0	0	0	0
Non-typed neurons	1	0	1	0
Orphan neurites	7	11	3	11
Gliaform cells	0	0	0	0
Blood capillaries	1	0	0	1
Total length (um) [2]	1112.5	911.7	1180.8	1375.8
Pyramidal process (um)	549.3	353.2	618.1	1052.2
Interneuron process (um)	0.0	0.0	0.0	0.0
Non-typed neuron process (um)	240.4	0.0	320.4	0.0
Orphan neurite (um)	173.5	558.4	242.3	291.4
Gliaform cell process (um)	0.0	0.0	0.0	0.0
Blood capillary (um)	149.3	0.0	0.0	32.2
Number of neurite segments	38	32	43	59
Neurite curvature (um^{-1}) [3]	0.41 (0.27)	0.43 (0.24)	0.28 (0.14)	0.32 (0.16)
Neurite radius (um) [3]	0.68 (0.43)	0.71 (0.48)	0.88 (0.68)	0.74 (0.56)
Number of spines	34	72	125	138
Spine curvature (um^{-1}) [3]	0.96 (0.45)	1.26 (0.51)	1.09 (0.53)	1.13 (0.57)
Spine radius (um) [3]	0.24 (0.09)	0.25 (0.08)	0.26 (0.08)	0.24 (0.08)
Spine length (um) [3]	1.06 (0.59)	1.07 (0.60)	1.32 (0.67)	1.07 (0.68)
Spine density (um^{-1}) [4]	0.066	0.131	0.141	0.139

[1] Image width x height x number of slices

[2] Spine length is not included.

[3] Mean (standard deviation)

[4] Spine density = number of spines / total length of spiny dendrite

S4 Table. Statistics of datasets and Cartesian coordinate models. (**G**) Control case N7.

Dataset name	N7A	N7B	N7C	N7D	N7E
Beamtime start date	2019.10.15	2019.10.15	2019.10.15	2019.6.7	2013.10.3
Image size (voxel) [1]	2080 x 2070 x 6997	2100 x 2070 x 6973	2110 x 2090 x 10276	1250 x 1300 x 4308	1660 x 1670 x 4911
Image size (um) [1]	101.3 x 100.8 x 340.8	102.3 x 100.8 x 339.6	102.8 x 101.8 x 500.4	56.3 x 58.5 x 193.9	66.7 x 67.1 x 197.4
Cortical depth of upper end (um)	2850	2240	2260	2230	2060
Number of model nodes	14774	7400	7989	1364	1609
Number of constituents	132	71	118	17	10
Pyramidal neurons	2	5	4	1	1
Interneurons	0	0	0	0	0
Non-typed neurons	0	1	0	0	0
Orphan neurites	129	65	114	16	9
Gliaform cells	1	0	0	0	0
Blood capillaries	0	0	0	0	0
Total length (um) [2]	8352.2	5716.3	5698.6	1007.8	888.2
Pyramidal process (um)	1888.2	2503.4	1754.6	641.8	592.8
Interneuron process (um)	0.0	0.0	0.0	0.0	0.0
Non-typed neuron process (um)	0.0	259.5	0.0	0.0	0.0
Orphan neurite (um)	5753.3	2953.4	3944.0	366.0	295.5
Gliaform cell process (um)	710.7	0.0	0.0	0.0	0.0
Blood capillary (um)	0.0	0.0	0.0	0.0	0.0
Number of neurite segments	217	163	174	44	31
Neurite curvature (um^{-1}) [3]	0.35 (0.25)	0.25 (0.19)	0.31 (0.25)	0.34 (0.21)	0.31 (0.33)
Neurite radius (um) [3]	0.60 (0.36)	0.89 (0.75)	0.72 (0.83)	0.70 (0.58)	0.87 (0.53)
Number of spines	1033	475	460	41	119
Spine curvature (um^{-1}) [3]	1.23 (0.58)	1.19 (0.61)	1.18 (0.58)	1.45 (0.74)	1.26 (0.54)
Spine radius (um) [3]	0.24 (0.07)	0.23 (0.07)	0.24 (0.07)	0.20 (0.08)	0.18 (0.06)
Spine length (um) [3]	1.26 (0.69)	1.33 (0.79)	1.25 (0.65)	0.80 (0.61)	1.52 (1.09)
Spine density (um^{-1}) [4]	0.163	0.114	0.128	0.101	0.193

[1] Image width x height x number of slices

[2] Spine length is not included.

[3] Mean (standard deviation)

[4] Spine density = number of spines / total length of spiny dendrite

S4 Table. Statistics of datasets and Cartesian coordinate models. (**G**) Control case N7 (cont'd).

Dataset name	N7F	N7G	N7H
Beamtime start date	2013.10.3	2013.10.3	2013.10.3
Image size (voxel) [1]	1660 x 1670 x 3835	1670 x 1680 x 3823	1660 x 1660 x 2753
Image size (um) [1]	66.7 x 67.1 x 154.2	67.1 x 67.5 x 153.7	66.7 x 66.7 x 110.7
Cortical depth of upper end (um)	1920	2320	2330
Number of model nodes	1296	645	785
Number of constituents	9	4	6
Pyramidal neurons	1	1	1
Interneurons	0	0	0
Non-typed neurons	0	0	0
Orphan neurites	8	3	5
Gliaform cells	0	0	0
Blood capillaries	0	0	0
Total length (um) [2]	723.8	584.7	364.8
Pyramidal process (um)	357.1	471.1	226.8
Interneuron process (um)	0.0	0.0	0.0
Non-typed neuron process (um)	0.0	0.0	0.0
Orphan neurite (um)	366.7	113.5	138.0
Gliaform cell process (um)	0.0	0.0	0.0
Blood capillary (um)	0.0	0.0	0.0
Number of neurite segments	24	20	14
Neurite curvature (um^{-1}) [3]	0.25 (0.28)	0.25 (0.21)	0.36 (0.33)
Neurite radius (um) [3]	1.28 (1.05)	1.33 (1.04)	0.87 (0.50)
Number of spines	138	52	53
Spine curvature (um^{-1}) [3]	1.35 (0.62)	1.33 (0.69)	1.37 (0.47)
Spine radius (um) [3]	0.19 (0.07)	0.24 (0.08)	0.21 (0.08)
Spine length (um) [3]	1.37 (0.72)	1.34 (0.78)	1.54 (0.97)
Spine density (um^{-1}) [4]	0.258	0.141	0.192

[1] Image width x height x number of slices

[2] Spine length is not included.

[3] Mean (standard deviation)

[4] Spine density = number of spines / total length of spiny dendrite

S4 Table. Statistics of datasets and Cartesian coordinate models. (**H**) Control case N8.

Dataset name	N8A	N8B	N8C	N8D	N8E
Beamtime start date	2019.10.15	2019.10.15	2019.10.15	2019.10.15	2019.10.15
Image size (voxel) [1]	2050 x 2050 x 6991	2060 x 2060 x 8632	2040 x 2050 x 5340	2070 x 2070 x 6981	2090 x 2080 x 6979
Image size (um) [1]	99.8 x 99.8 x 340.5	100.3 x 100.3 x 420.4	99.3 x 99.8 x 260.1	100.8 x 100.8 x 340.0	101.8 x 101.3 x 339.9
Cortical depth of upper end (um)	1400	1440	1680	1260	1120
Number of model nodes	2538	1400	4422	3605	1945
Number of constituents	37	14	62	55	35
Pyramidal neurons	3	3	4	7	1
Interneurons	1	0	0	0	0
Non-typed neurons	1	0	1	0	2
Orphan neurites	28	10	56	47	28
Gliaform cells	0	0	0	0	1
Blood capillaries	4	1	1	1	3
Total length (um) [2]	3393.6	1812.2	3397.8	4213.0	2138.0
Pyramidal process (um)	1380.4	1288.1	1381.8	2587.8	376.9
Interneuron process (um)	387.7	0.0	0.0	0.0	0.0
Non-typed neuron process (um)	143.9	0.0	53.9	0.0	88.8
Orphan neurite (um)	1181.7	456.3	1919.9	1597.8	1385.0
Gliaform cell process (um)	0.0	0.0	0.0	0.0	78.6
Blood capillary (um)	299.9	67.9	42.3	27.4	208.7
Number of neurite segments	77	33	108	137	55
Neurite curvature (um^{-1}) [3]	0.24 (0.15)	0.23 (0.14)	0.37 (0.19)	0.21 (0.15)	0.26 (0.22)
Neurite radius (um) [3]	1.01 (0.86)	1.33 (1.57)	0.69 (0.59)	1.05 (0.94)	1.09 (1.20)
Number of spines	27	68	60	99	65
Spine curvature (um^{-1}) [3]	1.24 (0.81)	0.95 (0.69)	1.04 (0.56)	1.01 (0.61)	0.95 (0.69)
Spine radius (um) [3]	0.26 (0.07)	0.29 (0.09)	0.25 (0.09)	0.27 (0.08)	0.25 (0.06)
Spine length (um) [3]	0.96 (0.53)	0.85 (0.44)	0.83 (0.45)	0.94 (0.51)	0.92 (0.44)
Spine density (um^{-1}) [4]	0.026	0.045	0.038	0.049	0.051

[1] Image width x height x number of slices

[2] Spine length is not included.

[3] Mean (standard deviation)

[4] Spine density = number of spines / total length of spiny dendrite

S4 Table. Statistics of datasets and Cartesian coordinate models. (**H**) Control case N8 (cont'd).

Dataset name	N8F	N8G
Beamtime start date	2019.10.15	2019.10.15
Image size (voxel) [1]	2050 x 2070 x 5336	2050 x 2040 x 6993
Image size (um) [1]	99.8 x 100.8 x 259.9	99.8 x 99.3 x 340.6
Cortical depth of upper end (um)	1160	970
Number of model nodes	1678	1188
Number of constituents	45	33
Pyramidal neurons	2	6
Interneurons	0	0
Non-typed neurons	3	0
Orphan neurites	37	26
Gliaform cells	0	0
Blood capillaries	3	1
Total length (um) [2]	2328.1	1592.2
Pyramidal process (um)	819.2	725.5
Interneuron process (um)	0.0	0.0
Non-typed neuron process (um)	240.6	0.0
Orphan neurite (um)	1019.0	813.0
Gliaform cell process (um)	0.0	0.0
Blood capillary (um)	249.3	53.7
Number of neurite segments	78	66
Neurite curvature (um^{-1}) [3]	0.25 (0.17)	0.26 (0.22)
Neurite radius (um) [3]	0.95 (0.63)	1.40 (1.46)
Number of spines	45	24
Spine curvature (um^{-1}) [3]	1.07 (0.67)	0.93 (0.62)
Spine radius (um) [3]	0.29 (0.08)	0.29 (0.09)
Spine length (um) [3]	0.93 (0.50)	0.97 (0.43)
Spine density (um^{-1}) [4]	0.056	0.040

[1] Image width x height x number of slices

[2] Spine length is not included.

[3] Mean (standard deviation)

[4] Spine density = number of spines / total length of spiny dendrite
